# Peptide presentation by bat MHC class I provides new insight into the antiviral immunity of bats

**DOI:** 10.1371/journal.pbio.3000436

**Published:** 2019-09-09

**Authors:** Dan Lu, Kefang Liu, Di Zhang, Can Yue, Qiong Lu, Hao Cheng, Liang Wang, Yan Chai, Jianxun Qi, Lin-Fa Wang, George F. Gao, William J. Liu

**Affiliations:** 1 NHC Key Laboratory of Medical Virology and Viral Diseases, National Institute for Viral Disease Control and Prevention, Chinese Center for Disease Control and Prevention, Beijing, China; 2 Faculty of Health Sciences, University of Macau, Macau SAR, China; 3 Institute of Biophysics, Chinese Academy of Sciences, Beijing, China; 4 Division of HIV/AIDS and Sex-transmitted Virus Vaccines, National Institutes for Food and Drug Control, Beijing, China; 5 Beijing Institutes of Life Science, University of Chinese Academy of Sciences, Beijing, China; 6 CAS Key Laboratory of Pathogenic Microbiology and Immunology, Institute of Microbiology, Chinese Academy of Sciences, Beijing, China; 7 Programme in Emerging Infectious Diseases, Duke-NUS Medical School, Singapore; University of Texas Southwestern Medical Center at Dallas, UNITED STATES

## Abstract

Bats harbor many zoonotic viruses, including highly pathogenic viruses of humans and other mammals, but they are typically asymptomatic in bats. To further understand the antiviral immunity of bats, we screened and identified a series of bat major histocompatibility complex (MHC) I Ptal-N*01:01–binding peptides derived from four different bat-borne viruses, i.e., Hendra virus (HeV), Ebola virus (EBOV), Middle East respiratory syndrome coronavirus (MERS-CoV), and H17N10 influenza-like virus. The structures of Ptal-N*01:01 display unusual peptide presentation features in that the bat-specific 3–amino acid (aa) insertion enables the tight “surface anchoring” of the P1-Asp in pocket A of bat MHC I. As the classical primary anchoring positions, the B and F pockets of Ptal-N*01:01 also show unconventional conformations, which contribute to unusual peptide motifs and distinct peptide presentation. Notably, the features of bat MHC I may be shared by MHC I from various marsupials. Our study sheds light on bat adaptive immunity and may benefit future vaccine development against bat-borne viruses of high impact on humans.

## Abstract 2

Bats harbor many viruses, including some that are highly pathogenic in humans and other mammals, but typically asymptomatic in bats. This study reveals the unusual mode of peptide presentation by bat MHC I and the landscape for its recognition by T-cell receptors, shedding light on the role of adaptive immunity in the ability of bats to coexist with viruses.

## Introduction

In recent years, emerging and re-emerging viral diseases with high mortality have continuously posed serious threats to human health [1–3]. In etiologic studies with convincing evidence, a series of such fatal diseases in humans have been confirmed or hypothesized to be caused by bat-borne viruses such as Hendra virus (HeV), Ebola virus (EBOV), Marburg virus, and coronaviruses, including severe acute respiratory syndrome coronavirus (SARS-CoV) and Middle East respiratory syndrome coronavirus (MERS-CoV) [4–10]. Similarly, fetal diseases in livestock have also been associated with emerging viruses of bat origin, such as the fatal disease outbreak of pigs in China, which was found to be caused by a novel coronavirus, swine acute diarrhea syndrome coronavirus (SADS-CoV), from bats [11,12]. Furthermore, it was shown that influenza-like viruses, termed H17N10 and H18N11 circulating among bats in Central America, may act as an ancient influenza reservoir [13,14]. It is well accepted now that bats harbor an exceptionally high proportion of zoonotic viruses with interspecies transmission potential [15] that have the potential to become virulent pathogens for humans. However, studies from wild or experimental bats indicate that most of these lethal viruses in humans and other mammals cause only asymptomatic infection in bats, suggesting a potentially special immune system in bats that is different from most other mammals [16,17].

Studies of comparative genomics and transcriptomics confirm that the critical components of the innate and adaptive immune system are conserved and functional in bats [18,19]. However, a large number of bat-unique characteristics related to immunity and antiviral responses have recently been identified. The most notable immune features involve the absence of key natural killer (NK) cell receptors and dampened cell signaling of type I interferons (IFNs) [18–20]. Meanwhile, stimulator of interferon genes (STING), an essential adaptor protein in multiple DNA sensing pathways, has a substitution in bats, compared with other mammals, that leads to decreased IFN activation [21]. Meanwhile, bats also possess special features to maintain an effective immune state. The genome analysis of *Rousettus aegyptiacus* revealed a dramatic difference from their functional gene counterparts in other mammals [19]. Indeed, bat IFNs and some IFN-stimulated genes are constitutively transcribed or maintain detectable expression levels in the absence of stimulation [22–24]. These characteristics may allow bats to fine-tune innate defense responses against insults by viral, bacterial, or host cytosolic DNA while avoiding excessive inflammation. However, the adaptive immune system in bats is less well studied.

Major histocompatibility complex (MHC) class I molecules (MHC I) present antigen peptides to the surface of antigen presenting cells (APCs) to active T cells through interaction with T-cell receptors (TCRs), which play pivotal roles in antiviral defense [25,26]. Epitopes or peptides are accommodated in the six pockets (A–F) of peptides/MHC I complexes, which were initially defined in humans [27]. Pocket A anchors the amine group of the amino terminal residue of the bound peptide, pocket B binds the side chain of peptide residue two, and pocket F accommodates the side chain of the carboxyl terminal residue [28]. In bats, the partial map of the *Pteropus alecto* MHC I region shows that MHC I genes are highly condensed and present within only one of the three highly conserved class I duplication blocks [29]. Genomic analyses of *R*. *aegyptiacus* demonstrate an expanded and diversified set of MHC I genes, with MHC I genes found outside the canonical region [19]. Sequences analyses, based on bat MHC I genes identified thus far, show that many of these bat MHC I molecules have a 3– or 5–amino acid (aa) insertion in the α1 domain compared with other mammals [20,29]. In the *R*. *aegyptiacus* genome, it is shown that 11 of the 12 MHC class I loci identified display the 3-aa insertion and only one locus without the insertion. Interestingly, one of the 11 MHC class I loci with 3-aa insertion is located in the canonical MHC alpha loci, indicating that both the MHC class I molecules with and without insertion can present the canonical binding surface [19]. Furthermore, the binding peptide motif of MHC I Ptal-N*01:01 derived from *P*. *alecto* has been identified. It displays a preference for peptides with Pro at their C terminus, which has never been seen in MHC I proteins of any other vertebrates [30]. However, the molecular basis for the peptide binding and presentation by bat MHC I remains unclear.

In this study, we screened bat MHC I Ptal-N*01:01–binding peptides from different bat-borne viruses (HeV, EBOV, MERS-CoV, and H17N10) and determined the structures of bat MHC class I complexed with these viral peptides. Unusual peptide presentation by bat MHC I with was demonstrated, which may help to understand the greater capacity of bats to coexist with a variety of viruses, from the perspective of adaptive immunity.

## Results

### Unusual characteristics of the bat MHC class I peptide binding groove

**Fig 1:**
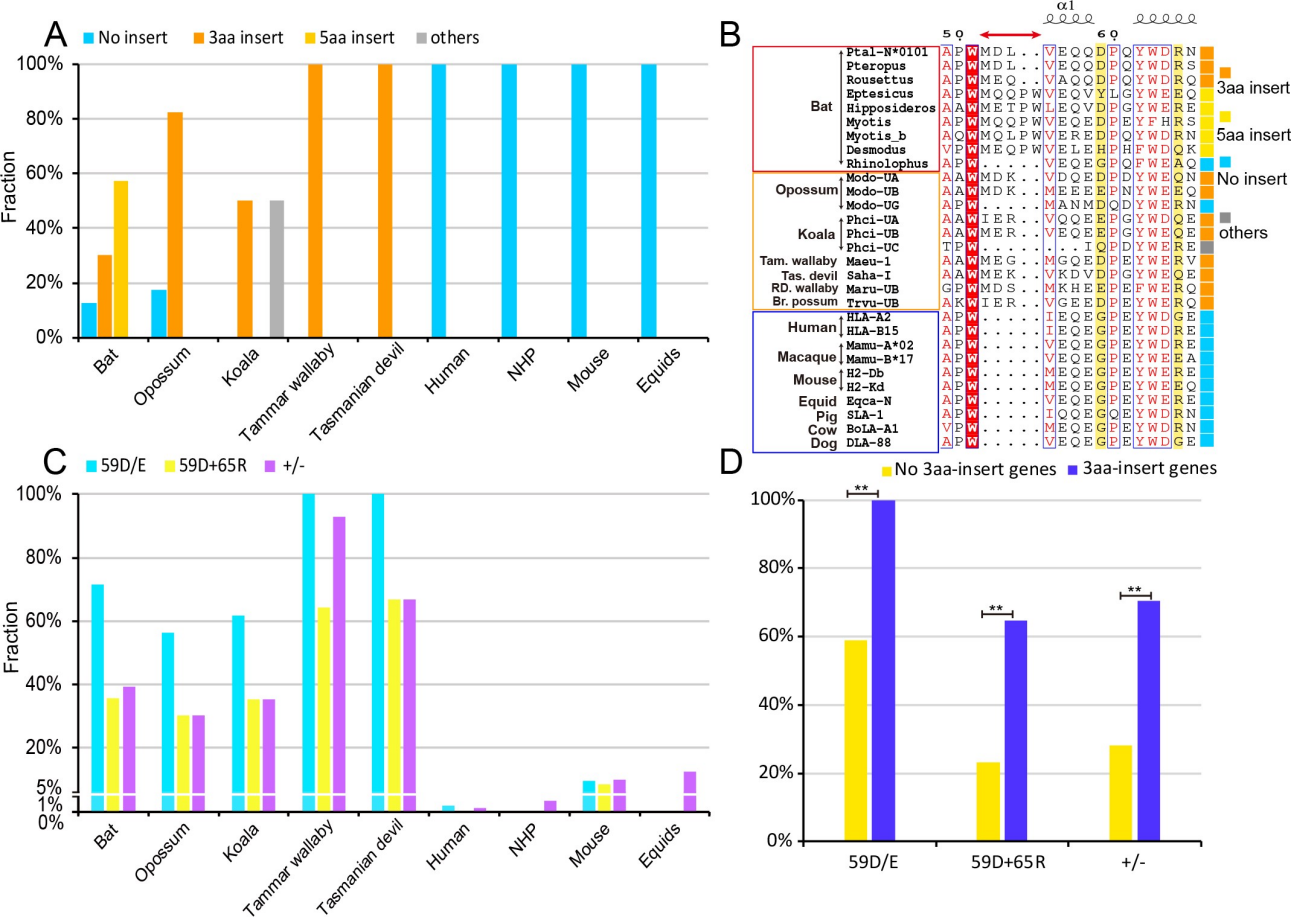
Unusual characteristics of bat MHC class I genes. (A) The proportions of MHC class I alleles with 3- or 5-aa insertions in bats, marsupials (opossum, koala, tammar wallaby, and Tasmanian devil), and higher mammals (human, NHP, mouse, and horse). The proportions of MHC class I alleles with the 3-aa insertion, 5-aa insertion, and no insertion are represented with orange, yellow, and cyan columns, respectively. The deletions (mainly including a 3-aa deletion) or insertions (mainly 1-aa insertions) other than the 3- and 5-aa insertions are termed as “others” in gray columns. The numerical data are included in S1 Data. (B) Structure-based sequence alignment of Ptal-N*01:01 and other representative (bats, marsupials, and higher mammals) MHC I molecules covering the residues from positions 49 to 66 (as in Ptal-N*01:01). The full information of the MHC I molecules of these species was listed in S1 Fig and S1 Table. Coils above the sequences indicated α-helices. Residues highlighted in red are completely conserved, and residues in blue boxes are highly (80%) conserved, with consensus amino acids in red. The residues at position 59 and 65 are shown in yellow. Special insertion positions in Ptal-N*01:01 are marked with red arrows above the sequences. The sequence alignment was generated with ClustalX and ESPript. To the right of the sequences, MHC class I alleles with the 3-aa insertion, 5-aa insertion, no insertion, and others are labeled with orange, yellow, cyan, and gray boxes, respectively. (C) The proportions of negatively charged residue Asp^59^ or Glu^59^ (“59D/E,” cyan columns), the pairing of the Asp^59^ and Arg^65^ (“D59+R65,” yellow columns), and the pairing of the negative-positive charged residues, termed as charge-matching at the two positions (“+/−,” purple columns) at the corresponding locations of MHC I in bats, marsupials, and higher mammals. (D) Statistical analysis of the 3-aa insertion and no 3-aa insertion alleles (no insertion and 5-aa insertion) in bats, respectively. Fisher exact test or the chi-squared test was used for the statistical analyses. **p < 0.01. aa, amino acid; MHC, major histocompatibility complex; NHP, nonhuman primate.

Previous work identified 56 bat MHC I genes from more than seven different species of bats on different continents. Each of these bat MHC I genes has the typical MHC I domains as in other mammals. However, examination of the retrieved set of bat MHC I sequences from GenBank revealed several unusual features. First, bat MHC I genes contain a 3- or 5-aa insertion within their peptide binding groove (PBG) compared with those from a variety of other mammals (i.e., between Trp^51^ and Ile^52^ of human HLA-A*0201) (Fig 1A and 1B). Within the bat MHC I genes, 30.36% possess a 3-aa insertion and 57.14% have the 5-aa insertion (Fig 1A, S1 Fig and S1 Table). The 5-aa insertion is unique to bat sequences, while the 3-aa insertion is present in both bats and some marsupials (82.61% of the opossum MHC I, 50% of the koala MHC I, and 100% of both tammar wallaby and Tasmanian devil MHC I contain the 3-aa insertion). All of the higher mammals, such as humans, nonhuman primates (NHPs), mouse, and horse, lack any insertion at this site (Fig 1A and S2A Fig).

Another feature of bat MHC I is the higher prevalence of a negatively charged residue at position 59 and a positively charged residue at position 65, as well as their pairing (according to the Ptal-N*01:01 residue code) (Fig 1B and S2A Fig). For the MHC I molecules of the higher mammals, the residue at the position corresponding to 59 (position 56 of human, NHP, mouse, and horse MHC I) is a highly conserved Gly. However, 71.43% of bat MHC Is have an Asp^59^ or Glu^59^ (59D/E) at this position (Fig 1C). The pairing of Asp^59^ and Arg^65^ (D59+R65) also occurs at a high fraction (35.71%) in bat MHC I compared with the MHC I of humans (0%), NHP (0%), mouse (0%), and horse (0%). Actually, the pairing of the charged residues at these two positions include different types: the negatively charged residues Asp/Glu at 59 paired with positively charged residues Arg/Lys at 65, and even positively charged residue Arg/Lys at 59 paired with Asp/Glu at 65 (as gene EPQ18390.1 in *Myotis brandtii*). Also, the pairing of the charged residues, termed as charge matching at the two positions (+/−), occurs at an even higher fraction for bat MHC I (39.29%). Interestingly, these features of bat MHC I are also prevalent in marsupials (Fig 1C).

To determine whether the insertion has any correlation with the unusual substitutions at positions 59/65, we analyzed the fractions of 59D/E, D59+R65, and the +/− charge matching in the bat MHC I genes with the 3-aa insertion. We found a significantly higher fraction of 59D/E (100%), D59+R65 (64.71%), and the +/− charge matching (70.59%) in the bat MHC I genes with the 3-aa insertion compared with the corresponding fractions of other bat MHC I genes (no insertion and 5-aa insertion) (59% for 59D/E, 23.08% for D59+R65, and 28.21% for the +/− charge matching) (Fig 1D). Collectively, these distinct features suggested an unusual PBG of bat MHC I, which may affect peptide binding and presentation.

### Ptal-N*01:01–binding peptides from known emerging viruses

**Fig 2:**
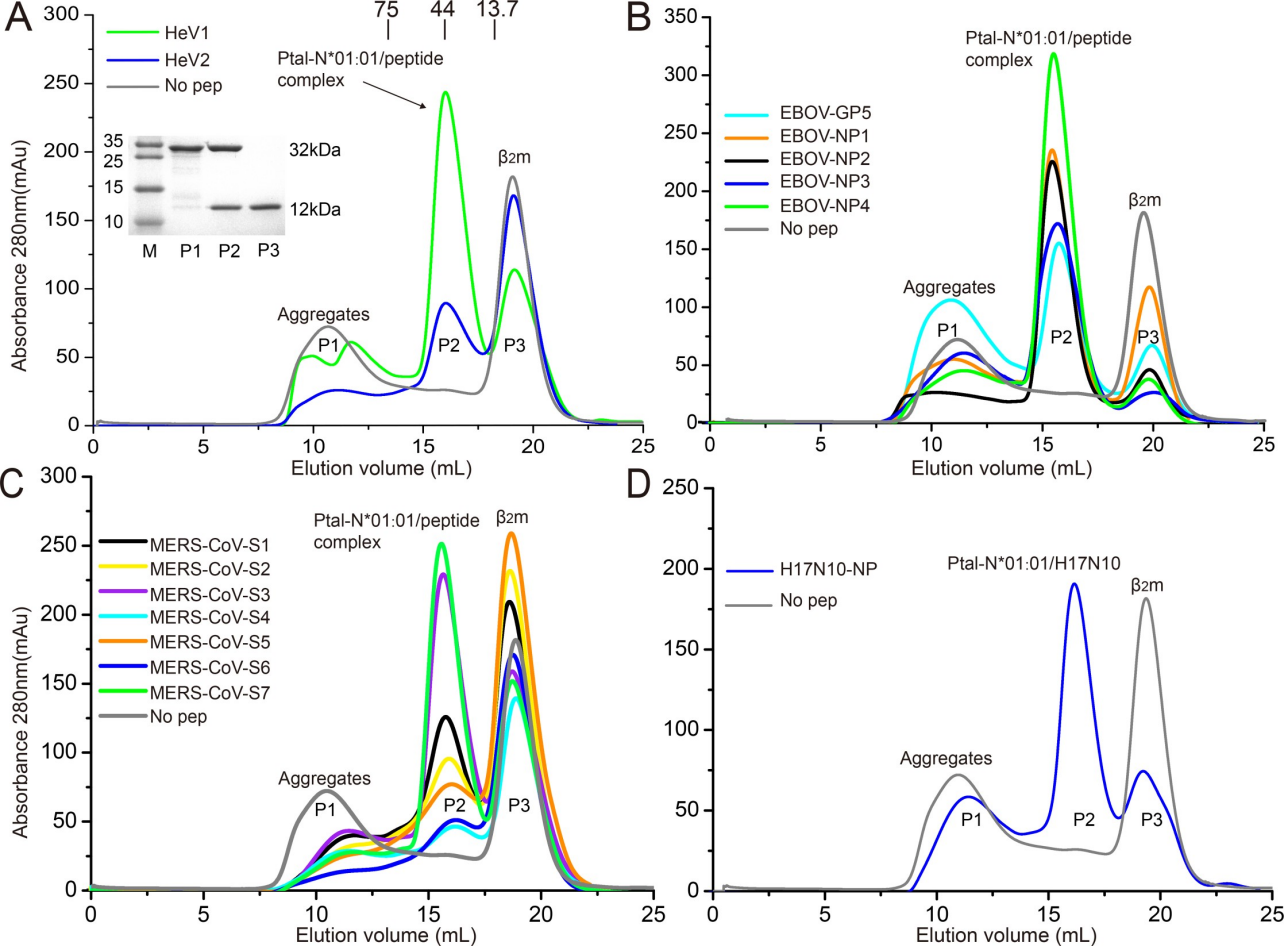
The identification of emerging and re-emerging virus-derived peptides binding to Ptal-N*01:01. The binding of peptides derived from HeV (A), EBOV (B), MERS-CoV (C), and H17N10 influenza-like virus (H17N10) (D) with Ptal-N*01:01 were evaluated by co-refolding. Co-refolding without any peptide was termed as the negative control (No pep), as curves in gray color. After properly refolding, the high-absorbance peaks of the correctly refolded MHC I with the expected molecular mass of 45 kDa were eluted at the estimated volume of 16 mL on a Superdex Increase 200 10/300 GL column. The profile is marked with the approximate positions of the molecular mass standards of 75.0, 44.0, and 13.7 kDa. Inset, reduced SDS–PAGE gel (15%) of Ptal-N*01:01/HeV1 complex for peak 1 (P1), peak 2 (P2), and peak 3 (P3). Lane M contains molecular-mass markers (labeled in kDa). P1, P2, and P3 represent the aggregated heavy chain, the correctly refolded heterotrimer Ptal-N*01:01 complex (45 kDa), and the extra β_2_m, respectively. β_2_m, β_2_-microglobulin; HeV, Hendra virus; EBOV, Ebola virus; MERS-CoV, Middle East respiratory syndrome coronavirus; MHC, major histocompatibility complex; P1, peak 1; P2, peak 2; P3, peak 3.

To verify the peptide binding motif of Ptal-N*01:01 and to screen the potential bat MHC I T-cell epitopes from recently emerging and re-emerging viruses with bats as their potential reservoir, we predicted Ptal-N*01:01–binding peptides from EBOV, MERS-CoV, and H17N10/H18N11 (S2 Table). The previously determined Ptal-N*01:01–binding peptides derived from HeV were also included as a positive control [30]. The binding capacity of these peptides were evaluated by their ability to facilitate the in vitro renaturation of Ptal-N*01:01. Generally, the peptides with higher binding capability to MHCs would have a higher production of the heterotrimer MHC complexes but lower production of β_2_-microglobulin (β_2_m). Seven peptides from MERS-CoV, five from EBOV, one from H17N10, and the two HeV-derived peptides helped Ptal-N*01:01 naturally refold (Fig 2). The lengths of the Ptal-N*01:01–binding peptides cover a range from octamers to tridecamer. These peptides possess an Asp at the P1 position (with Gln in one peptide: MERS-CoV-S4), an aromatic aa (Phe or Tyr) at the P2 position, and a Pro or Leu at the PΩ position (C terminus of the peptide) (S2 Table).

### The structures of Ptal-N*01:01 verify the motifs of the binding peptides derived from pathogens

The binding peptide motif of bat MHC I Ptal-N*01:01, with the negative-charged Asp at the P1 position and a Pro at the PΩ position, is indeed extremely rare in class I binding peptide repertoires of humans and other mammals. To verify the uncommon peptide presentation features of bat MHC I, we determined the crystal structures of Ptal-N*01:01 in complex with the two HeV-derived peptides, HeV1 (DFANTFLP) and HeV2 (DYINTNVLP), two EBOV-derived peptides, EBOV-NP1 (DFQESADSFL) and EBOV-NP2 (DFQESADSFLL), one H17N10-derived peptide, H17N10-NP (DFEKEGYSL), and one MERS-CoV-derived peptide, MERS-CoV-S3 (DFTCSQISP) (Table 1).

**Table 1:** X-ray data processing and refinement statistics.

Parameter	Ptal-N*01:01/HeV1	Ptal-N*01:01(-3aa)/ HeV1	Ptal-N*01:01/HeV2	Ptal-N*01:01/H17N10-NP	Ptal-N*01:01/EBOV-NP1	Ptal-N*01:01/EBOV-NP2	Ptal-N*01:01/MERS-CoV-S3	Ptal-N*01:01/HeV1 (hβ_2_m)	Bat β_2_m
PDB code	6J2D	6J2H	6J2F	6J2I	6J2E	6J2G	6J2J	6K7T	6K7U
Peptide sequence	DFANTFLP	DFANTFLP	DYINTNVLP	DFEKEGYSL	DFQESADSFL	DFQESADSFLL	DFTCSQISP	DFANTFLP	
Data Processing									
Space group	C2221	C2221	C2221	C2221	C2221	C2221	C2221	P21212	C2221
Cell parameters (Å)									
a (Å)	a = 72.458	a = 100.651	a = 100.245	a = 100.937	a = 100.740	a = 101.397	a = 100.035	a = 73.880	a = 33.710
b (Å)	b = 86.272	b = 102.504	b = 102.527	b = 104.641	b = 102.596	b = 103.307	b = 102.714	b = 88.770	b = 114.640
c (Å)	c = 134.728	c = 178.505	c = 176.312	c = 180.782	c = 177.625	c = 180.057	c = 177.674	c = 67.170	c = 57.310
α (°)	α = 90.0	α = 90.0	α = 90.0	α = 90.0	α = 90.0	α = 90.0	α = 90.0	α = 90.0	α = 90.0
β (°)	β = 90.0	β = 90.0	β = 90.0	β = 90.0	β = 90.0	β = 90.0	β = 90.0	β = 90.0	β = 90.0
γ (°)	γ = 90.0	γ = 90.0	γ = 90.0	γ = 90.0	γ = 90.0	γ = 90.0	γ = 90.0	γ = 90.0	γ = 90.0
Wavelength (Å)	0.97852	0.97918	0.97900	0.97918	0.97915	0.97776	0.97853	0.97915	0.97776
Resolution (Å)	50.0–2.3	50.0–2.3	50.0–1.9	50.0–2.03	50.0–2.1	50.0–2.4	50.0–2.5	50.0–1.6	50.0–1.6
Total reflections	125,607	479,029	969,116	495,959	534,807	351,332	193,609	579,888	112,247
Unique reflections	18,451	40,730	72,225	42,195	54,243	36,391	31,557	58,687	15,045
Completeness (%)^a^	99.9 (100.0)	98.0 (95.6)	100 (100.0)	99.7 (99.9)	100 (100.0)	99.2 (98.1)	97.2 (95.5)	99.5 (100.0)	100 (100.0)
Redundancy	6.8 (7.0)	11.8 (12.0)	13.4 (13.5)	11.8 (11.9)	9.9 (6.8)	9.7 (10.0)	6.1 (5.9)	9.9 (9.8)	7.5 (7.6)
*R*_merge_ (%)^b^	18.5 (76.4)	11.9 (104.5)	6.3 (82.9)	10.5 (170.6)	11.4 (99.7)	11.9 (99.7)	14.5 (131.7)	7.0 (58.0)	6.5 (86.0)
*I/σ*	4.2 (2.2)	57.1 (3.8)	1.3 (3.3)	28.1 (1.6)	69.3 (2.4)	10.1 (2.0)	3.7 (1.2)	31.2 (4.2)	2.7 (2.3)
Refinement									
*R*_work_ (%)^c^	22.1	19.7	20.3	21.4	20.9	20.9	19.4	21.1	19.6
*R*_free_ (%)	28.2	22.8	23.1	25.3	25.9	24.1	26.7	23.4	20.2
RMSD^d^									
Bonds (Å)	0.008	0.007	0.010	0.006	0.008	0.005	0.010	0.009	0.006
Angle (°)	1.055	0.756	1.135	0.730	1.180	0.750	1.250	0.980	1.010
Average B factor (Å^2^)	42.94	34.05	18.24	37.54	29.28	43.92	43.10	20.53	19.61
Ramachandran plot quality (%)									
Favored (%)	97.34	99.07	98.02	98.17	96.84	95.93	94.62	97.67	96.84
Allowed (%)	2.66	0.93	1.98	1.83	3.16	4.07	5.38	2.33	3.16
Outliers (%)	0.00	0.00	0.00	0.00	0.00	0.00	0.00	0.00	0.00

^a^Values in parentheses refer to statistics in the outermost resolution shell.

^b^Data completeness = (number of independent reflections)/(total theoretical number).

^c^*R*_merge_ = ∑_*hkl*_∑_*i*_|*I*_*i*_−〈*I*〉|∑_*hkl*_∑_*i*_*I*_*i*_ , where *I*_*i*_ is the observed intensity, and 〈*I*〉 is the average intensity of multiple observations of symmetry related reflections.

^d^*R* = ∑_*hkl*_‖*F*_*obs*_|−*k*|*F*_*call*_‖/∑_*hkl*_|*F*_*obs*_|, where *R*_free_ is calculated for a randomly chosen 5% of reflections, and *R*_work_ is calculated for the remaining 95% of reflections used for structure refinement.

^e^Ramachandran plots were generated by using the PROCHECK program of the CCP4i suite.

Abbreviations: aa, amino acid; β_2_m, β2-microglobulin; EBOV, Ebola virus; hβ_2_m, human β_2_-microglobulin; HeV, Hendra virus; MERS-CoV, Middle East respiratory syndrome coronavirus; PDB, Protein Data Bank; RMSD, root mean square deviation

**Fig 3:**
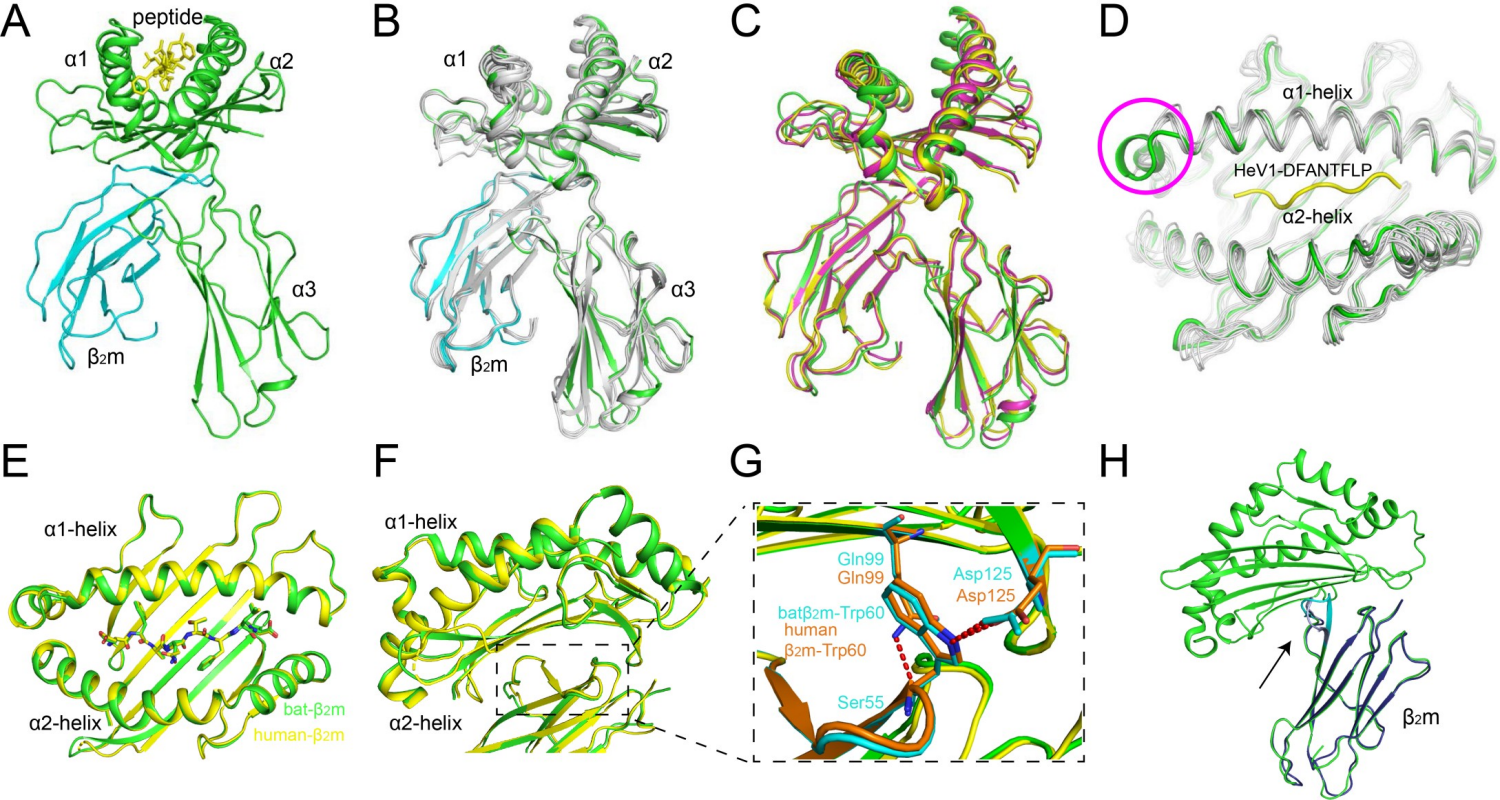
Overview of Ptal-N*01:01/peptide structures. (A) Overview of the structure of the Ptal-N*01:01/HeV1. The peptide HeV1 is presented as yellow sticks in the peptide-binding cleft. The heavy chain of Ptal-N*01:01 and bat β_2_m are shown as green and cyan cartoons, respectively. (B) The superimposition of different Ptal-N*01:01 complexes was performed using the determined structures: Ptal-N*01:01/HeV1, Ptal-N*01:01/HeV2, Ptal-N*01:01/EBOV-NP1, Ptal-N*01:01/EBOV-NP2, Ptal-N*01:01/H17N10-NP, and Ptal-N*01:01/MERS-CoV-S3. The peptides were omitted. (C) Structural alignment of MHC I heavy chains and β_2_m exhibited a similar overall conformation of Ptal-N*01:01 (green, peptide HeV1), HLA-A*0201 (yellow, PDB code: 3I6G), and H-2K^d^ (purple, PDB code: 5GR7). (D) The superimposition of the α1α2 domains of Ptal-N*01:01 (green) with other vertebrate MHC I molecules: human HLA-A*0201 (3I6G), macaque Mamu-A*02 (3JTT), swine SLA-1*0401 (3QQ4), equine Eqca-N*00602 (4ZUU), bovine N*01801 (3PWV), canine DLA-88*50801 (5F1I), and murine H2-K^d^ (5GR7) (all in white). The distinct conformations at the N terminus between the α1-helices of Ptal-N*01:01 (green) and the MHC I from other vertebrates (white) are labeled by a pink circle. The peptide HeV1 in Ptal-N*01:01 is presented in yellow loops. (E) The structural alignment of Ptal-N*01:01 (peptide HeV1) when renatured with bat β_2_m (green) and human β_2_m (yellow). HeV1 showed similar conformation in the binding groove. (F) The black box in the dashed line indicates the similar conformations of residues in the interface of the bat (green) and human β_2_m (yellow) binding to α1α2 domains of Ptal-N*01:01 heavy chain. (G) Similar binding of Ptal-N*01:01 heavy chain to bat and human β_2_m. Trp60 in bat β_2_m (cyan) and human β_2_m (orange) binds to Gln99 and Asp125 in Ptal-N*01:01 when forming a complex. (H) The alignment of monomer bat β_2_m (blue) with HeV-1/Ptal-N*01:01-batβ_2_m (green) complexes. Resides Ser55 to Tyr63 in monomer bat β_2_m (light blue) shift minorly after forming a complex (cyan). β_2_m, β_2_-microglobulin; EBOV, Ebola virus; HeV, Hendra virus; MERS-CoV, Middle East respiratory syndrome coronavirus; MHC, major histocompatibility complex; PDB, Protein Data Bank.

The overall structures of Ptal-N*01:01 display the common characteristics of classical MHC I molecules in other mammals, with the extracellular region of the heavy chain folding into three different domains. The α1 and α2 domains construct a typical PBG that contains two α1-helices and eight β-sheets, and the α3 domain and β_2_m display typical immunoglobulin (Ig) domains and underpin the peptide binding domain (Fig 3A). The all-atoms superimposition of Ptal-N*01:01/HeV1 onto the other five structures demonstrates a similar overall conformation, with root mean square deviations (RMSDs) of 0.344–0.517 Å (Fig 3B). The superimposition of Ptal-N*01:01/HeV1 onto human MHC I HLA-A2 and mouse MHC I H-2K^d^ generated RMSDs of 1.198 and 1.119 Å, respectively (Fig 3C). The most distinct differences between Ptal-N*01:01 and the MHC I from other vertebrates are located in the N terminus of the PBG, with an extension of the α1-helix in Ptal-N*01:01 (Fig 3D).

To elucidate whether the peptide-presenting features of a bat MHC I molecule can be influenced by binding to human β_2_m, we solved the structure of the Ptal-N*01:01 heavy chain complexed with human β_2_m (Ptal-N*01:01-h) at a resolution of 1.6 Å (Table 1). Comparing HeV1/Ptal-N*01:01 renatured with bat β_2_m and human β_2_m, the structural conformations of both HeV1 peptides in the two structures are also similar, with an RMSD of 0.228 Å in the two binding grooves (Fig 3E). In addition, the overall structures are quite similar, with the RMSD of 0.564 Å of all atoms (Fig 3F). And the key residues binding to α1α2 domains and α3 domain of the Ptal-N*01:01 heavy chain are highly conserved in human and bat β_2_m (S2B Fig). Further analysis shows Trp60 in bat β_2_m binds to Gln99 and Asp125 in Ptal-N*01:01 when forming a complex, which is also conserved in human β_2_m when binding to Ptal-N*01:01 (Fig 3G). It indicates that the structure of the peptide loaded in the groove of Ptal-N*01:01 was not affected by the substitution of the β_2_m subunit. We also determined the structure of monomer bat β_2_m without MHC I (Table 1). The structure alignment of bat β_2_m monomer with β_2_m subunit in MHC complexes indicates a minor conformational shift of the loop resides Ser55 to Tyr63 in bat β_2_m after forming a complex (Fig 3H).

**Fig 4:**
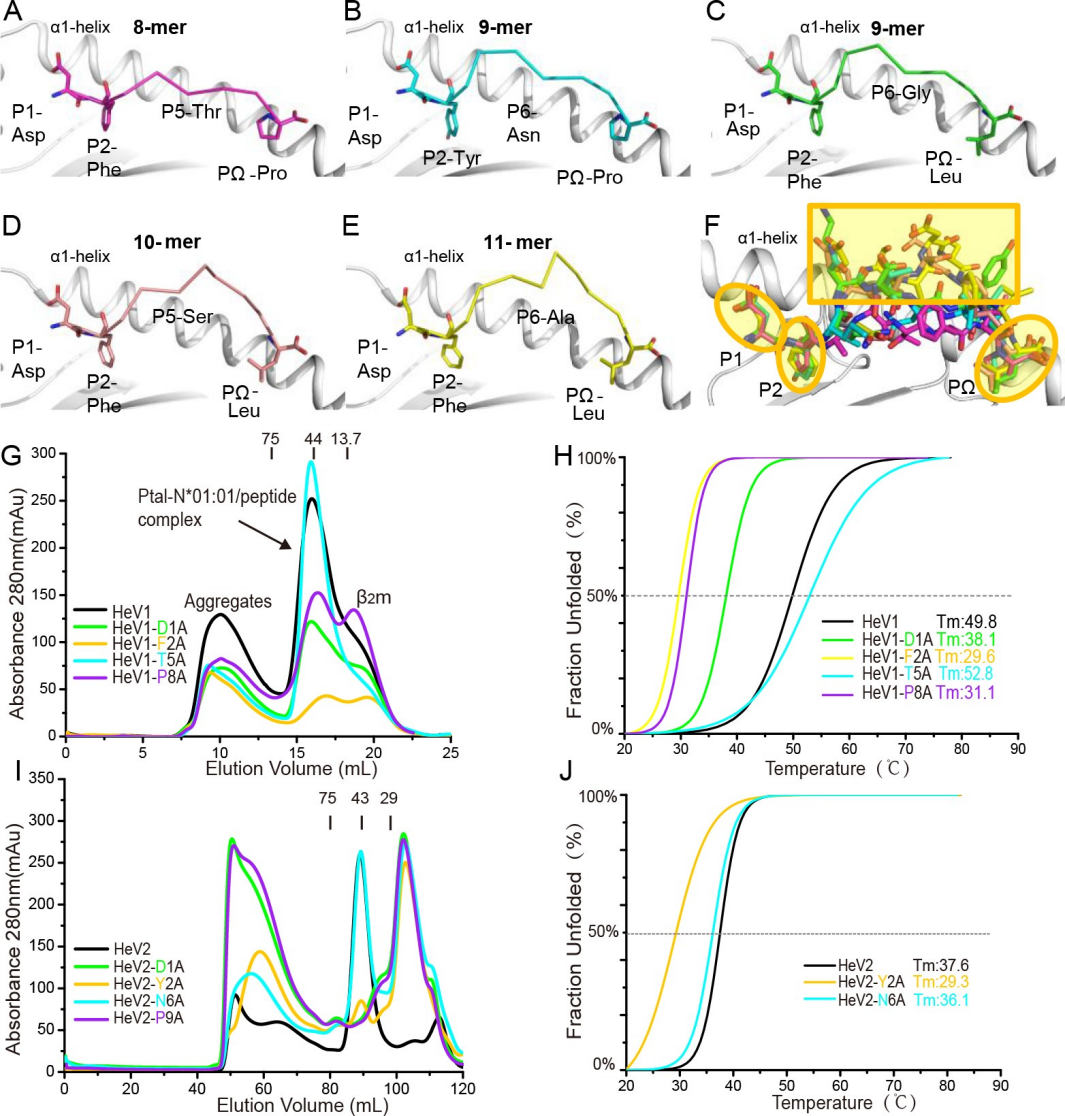
The structural conformation and motif of the binding peptides presented by Ptal-N*01:01. (A-E) The overall conformation of Ptal-N*01:01–binding peptides HeV1 (A) and HeV2 (B) from HeV, H17N10-NP (C) from H17N10 influenza-like virus, and EBOV-NP1 (D) and EBOV-NP2 (E) from EBOV. (F) The superimposition of presented peptides HeV1, HeV2, H17N10-NP, EBOV-NP1, and EBOV-NP2 by Ptal-N*01:01. The peptides are aligned according to the superimposition of the α1 and α2 domains of the five structures of Ptal-N*01:01. The conserved conformations of residues in the P1, P2, and PΩ positions are shown in yellow ellipses. Most conformational distinctions are located in the central region of the peptides, marked by a yellow rectangle. (G-J) The capacity of HeV1 (G), HeV2 (I), and their Ala substitutions for binding to Ptal-N*01:01 was evaluated by in vitro refolding. The thermostabilities of Ptal-N*01:01 with HeV1 (H) and HeV2 (J) peptides and their Ala substitutions were tested by circular dichroism (CD) spectroscopy. The numerical data are included in S1 Data. The curves for the unfolded fractions were determined by monitoring the CD value at 218 nm. The temperature was increased by 1°C/minute. Shown are the fitting data to the denaturation curves using the Origin 8.0 program (OriginLab). The *T*_m_s of different peptides are indicated by the dashed gray lines at the 50% fraction unfolded. β_2_m, β_2_-microglobulin; CD, circular dichroism; EBOV, Ebola virus; HeV, Hendra virus; *T*_m_, midpoint transition temperature.

Although having different lengths, the 8-mer peptide HeV1, 9-mers HeV2 and H17N10-NP, 10-mer EBOV-NP1, and 11-mer EBOV-NP2 in the PBG of Ptal-N*01:01 all display an M-shaped conformation, with P2 and PΩ residues as the primary anchors and the P5 or P6 residue as the secondary middle anchors (Fig 4A–4F and S3A–S3E Fig). The P1 Asp of all of the peptides adopts a rigid conformation upward to the α1-helix of the heavy chain. In the structure of the Ptal-N*01:01/MERS-CoV-S3 complex, the conformation of the P3–P8 residues of the peptide could not be determined due to their poor electron densities (S3F Fig), revealing a flexible conformation in the middle region of this MERS-CoV-S3 peptide. However, the other three residues (P1-Asp, P2-Phe, and PΩ-Pro) with electron densities available adopt similar conformations as in the other five structurally determined peptides. As a 9-mer peptide, MERS-CoV-S3 possesses a Glu at P6, corresponding to the P6-Asn in 9-mer HeV2, which is the secondary anchor residue. The larger Glu may not be able to locate in the PBG, which leads to a flexible conformation of the MERS-CoV-S3.

To further validate the role of the unusual binding peptide motif of bat MHC I Ptal-N*01:01, Ala mutations at residues P1, P2, PΩ, and the middle (P5 or P6) positions of peptides HeV1 and HeV2 were evaluated by refolding assays (Fig 4G and 4I). The thermal stability of the resulting heterotrimers was monitored by circular dichroism (CD) spectroscopy (Fig 4H and 4J, S3 Table). Substitution of Ala at P1 (HeV2-D1A), P2 (HeV2-Y2A), and P9 (HeV2-P9A) of peptide HeV2 led to nearly no refolding for Ptal-N*01:01, whereas Ala at P1 (HeV1-D1A), P2 (HeV1-F2A), and P8 (HeV1-P8A) of peptide HeV1 still supported refolding but with significantly lower stability than the original peptide. In contrast, substitution of Ala at P5 of HeV1 (HeV1-T5A) and at P6 of HeV2 (HeV1-N6A) led to similar yield of refolded heterotrimers, indicating a similar stability compared with the original peptides. Thus, through these analyses, the P1 anchor of Ptal-N*01:01–binding peptides seems to be as significant as the P2 and PΩ anchors.

### The insertion induces uncommon peptide P1 anchoring in bat MHC I

**Fig 5:**
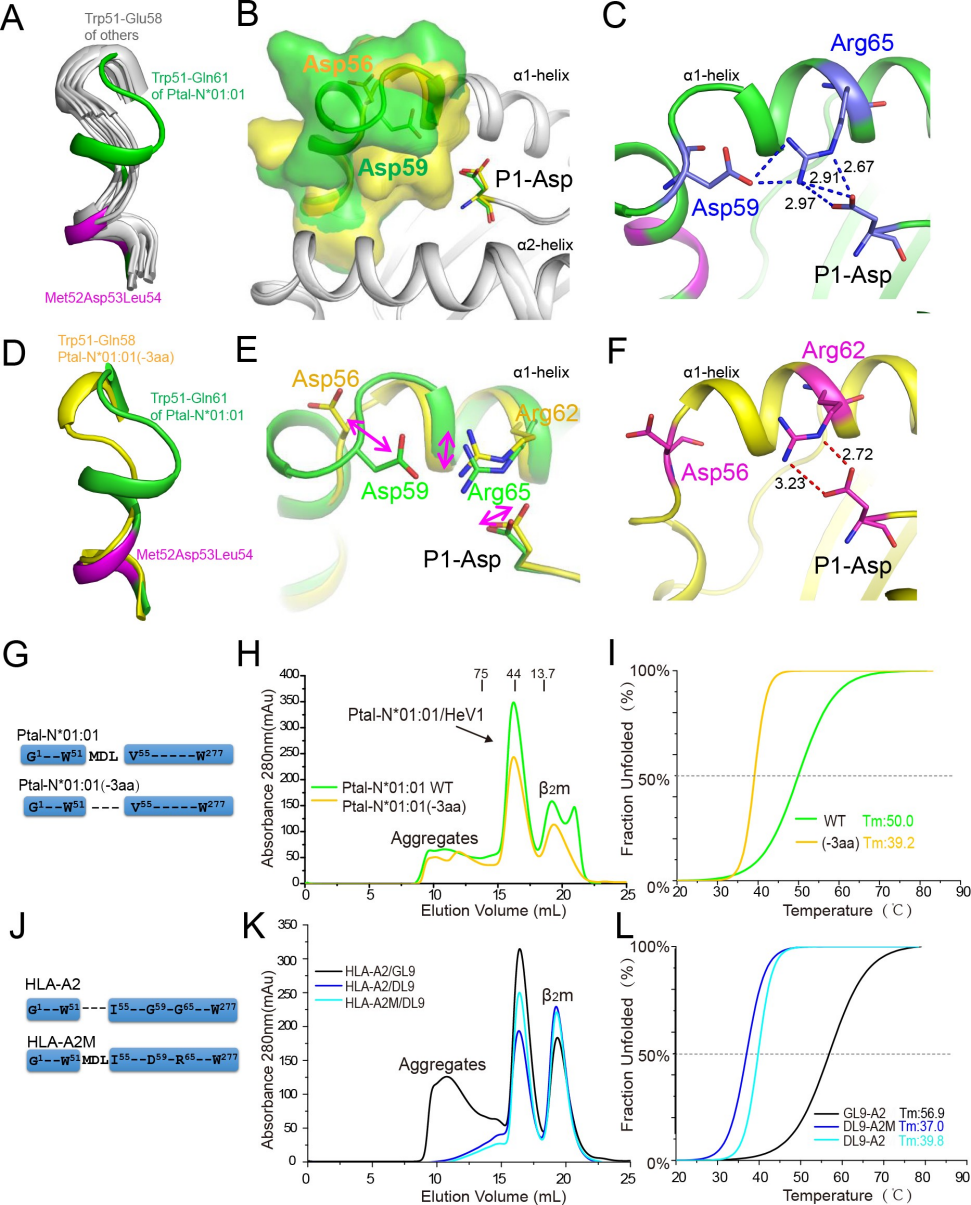
The 3-aa (Met^52^Asp^53^Leu^54^) insertion contributes an uncommon P1 anchoring of bat MHC I. (A) The structural superimposition of the N terminus of PBGs from different MHC I molecules. Trp^51^–Gln^61^ of Ptal-N*01:01 in green and structurally corresponding residues Trp^51^–Glu^58^ of HLA-A*0201 (3I6G), macaque Mamu-A*02 (3JTT), swine SLA-1*0401 (3QQ4), equine Eqca-N*00602 (4ZUU), bovine N*01801 (3PWV), canine DLA-88*50801 (5F1I), and murine H2-K^d^ (5GR7) in white. The 3-aa Met^52^Asp^53^Leu^54^ insertion in bat MHC I is in purple. (B) The structural superimposition of Ptal-N*01:01 and its mutant Ptal-N*01:01(-3aa) (with Met^52^Asp^53^Leu^54^ deletion). The residues Trp^51^–Gln^61^ of Ptal-N*01:01 (green) and Trp^51^–Gln^58^ of Ptal-N*01:01(-3aa) (yellow) are presented in surface representation. The heavy chains of these two molecules are shown as white cartoons. The P1 positions of the peptides are shown as sticks. (C) The hydrogen bond network in the A pocket of Ptal-N*01:01/HeV1. Residues Asp^59^, Arg^65^, and P1-Asp of HeV1 are shown as blue sticks and form a triangular network of hydrogen bonds in blue dashed lines. (D) Different conformations of the N terminus of the α1-helices of Ptal-N*01:01 (green) and mutant Ptal-N*01:01(-3aa) (yellow). (E) The conformational shift of Asp, Arg, and P1-Asp of HeV1 in the structures Ptal-N*01:01/HeV1 (green) and Ptal-N*01:01(-3aa)/HeV1 (yellow), as indicated by purple arrows. (F) The N-terminal conformation of the α1-helix and hydrogen bonds (red dashed lines) within the structure of mutant Ptal-N*01:01(-3aa)/HeV1. Asp, Arg, and HeV1 P1-Asp are shown as purple sticks. (G) Schematic diagram of the construction of the mutant Ptal-N*01:01(-3aa) with a Met^52^Asp^53^Leu^54^ deletion compared with the wild-type Ptal-N*01:01. (H-I) The capabilities of peptide HeV1 presented by Ptal-N*01:01 and mutant Ptal-N*01:01(-3aa) were evaluated by in vitro refolding (H) and CD spectroscopy (I). The numerical data are included in S1 Data. (J) Schematic diagram of the construction of the mutant HLA-A2M with a Met^52^Asp^53^Leu^54^ insertion and D59+R65 compared with the wild-type HLA-A2. (K-L) The capabilities of peptide DL9 (the first amino acid of GL9 change to D) presented by HLA-A2 and mutant HLA-A2M were evaluated by in vitro refolding (K) and CD spectroscopy (L). aa, amino acid; β_2_m, β_2_-microglobulin; CD, circular dichroism; DL9, DILGFVFTL; GL9, GILGFVFTL; HeV, Hendra virus; MHC, major histocompatibility complex; PBG, peptide binding groove; P1, position 1; *T*_m_, midpoint transition temperature; WT, wild type.

Bat Ptal-N*01:01 possesses the Met-Asp-Leu insertion within the N terminus of its α1-helix (between residues 51 and 52 of HLA-A*0201). Compared with the structures available for the MHC I of other mammals, such as humans and mouse, the Ptal-N*01:01 structure displays an extension of the α1-helix of the PBG (Fig 5A). The 3-aa insertion pushes residue Asp^59^ closer to the N terminus of the binding peptide, which leads to the extension of the negatively charged side chain of Asp^59^ into the PBG (Fig 5B). The bat MHC I residue Asp^59^ participates in the formation of the A pocket (S4 Fig). Detailed analysis indicated that Asp^59^, Arg^65^, and the P1-Asp of the peptides in all six structures of Ptal-N*01:01 form a triangular network of hydrogen bonds (Fig 5C and S5 Fig).

To further investigate the role of the 3-aa insertion in the peptide binding and presentation of Ptal-N*01:01, we constructed the Ptal-N*01:01(-3aa) mutant, which deleted the 3-aa insertion (Fig 5G). The structure of Ptal-N*01:01(-3aa)/HeV1 displayed a shortened α1-helix that is similar to the human HLA-A*0201 (Fig 5D). Meanwhile, although the salt bridge was still observed between Arg^65^ of Ptal-N*01:01 and the P1-Asp, both the Arg^65^ and P1-Asp displayed a conformational shift (Fig 5E). The triangular network of the hydrogen bonds between Asp^59^ (Residue Asp^56^ in the mutant), Arg^65^ (Residue Arg^62^ in the mutant), and the P1-Asp of HeV1 within the structure of the wild-type Ptal-N*01:01 was broken (Fig 5F). One of the hydrogen bonds between the two residues Arg^65^ (Residue Arg^62^ in the mutant) and the P1-Asp of HeV1 was lost, and the remaining two hydrogen bonds extended from 2.97 to 3.23 Å and 2.67 to 2.72 Å, respectively.

We also investigated whether the 3-aa deletion of Ptal-N*01:01 influenced binding ability to the peptides. Ptal-N*01:01(-3aa) was still renatured in the presence of peptide HeV1 but with a much lower yield of the heterotrimer complex (Fig 5H). CD spectroscopy also indicated a weaker binding of Ptal-N*01:01(-3aa) with the peptide (Fig 5I). To further verify the unusual peptide presentation and preference for peptides with a P1-Asp in bat Ptal-N*01:01, we constructed the HLA-A2M mutant, which has a 3-aa insertion and the charge matching residues at positions 59/65 based on HLA-A*02:01(Fig 5J). Both the refolding assay and CD spectroscopy indicated a stronger binding of DL9 (G1D mutant at P1 of peptide GL9) with the HLA-A2M compared with HLA-A*02:01, and the HLA-A*02:01 has a higher binding capacity to GL9 than DL9 (Fig 5K and 5L).

### The unusual preference of Pro as the PΩ anchor of Ptal-N*01:01–binding peptides

**Fig 6:**
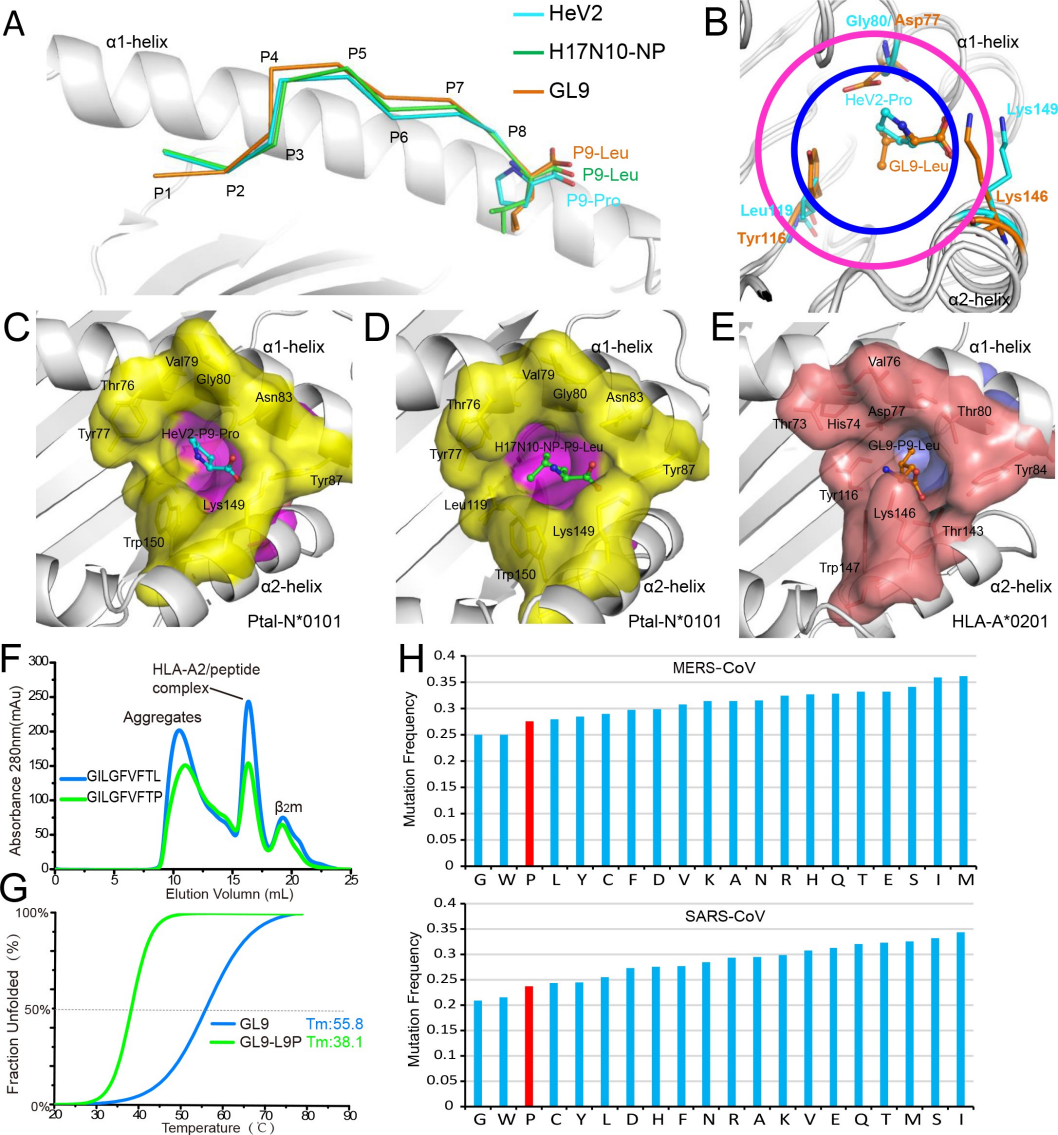
The extraordinary preference for Pro as the PΩ anchor of Ptal-N*01:01–binding peptides. (A) The superimposition of 9-mer peptides HeV2 (cyan) and H17N10-NP (green) presented by Ptal-N*01:01 and GL9 (orange) presented by HLA-A*0201 (PDB code: 3I6G). The heavy chains are represented as gray cartoons. The PΩ anchors of peptides are shown as sticks, and the side chains of other residues of the peptides are omitted. (B) Distinct F pockets of Ptal-N*01:01 and HLA-A*0201. The heavy chains are represented as gray loops. Gly^80^, Leu^119^, and Lys^149^ of Ptal-N*01:01 are represented as cyan sticks. The corresponding residues of HLA-A*0201 are shown as orange sticks. The given entrances for F pockets of Ptal-N*01:01 and HLA-A*0201 are labeled by purple and deep blue circles, respectively. (C-D) The side chains of PΩ-Pro from peptide HeV2 (C) and PΩ-Leu from peptide H17N10-NP (D) insert into the F pockets of Ptal-N*01:01. The F pocket of Ptal-N*01:01 is indicated in surface representation with a yellow entrance and purple bottom. (E) The side chain of residue PΩ-Leu from peptide GL9 inserts into the F pocket of HLA-A*0201. The F pocket of HLA-A*0201 is indicated by the representation of a brown entrance with a light blue bottom. (F-G) The stabilities of HLA-A*0201 complexed with peptide GL9 and its mutant GL9-L9P (with a Pro at the P9 position) were evaluated by in vitro refolding (F) and CD spectroscopy (G). The numerical data are included in S1 Data. (H) The relatively lower mutation frequency of amino acid Pro (red bar) as Try and Gly among the 20 component amino acids in the proteins of MERS-CoV and SARS-CoV. The mutation frequency = the number of overall mutations for each amino acid/(the number of occurrences of the amino acid in the reference sequence×total number of sequences). The proteomes of 1,000 MERS-CoV genomes and SARS-CoV genomes were retrieved from GenBank, respectively. The detailed information is included in S2 Data. CD, circular dichroism; GL9, GILGFVFTL; HeV, Hendra virus; MERS-CoV, Middle East respiratory syndrome coronavirus; PDB, Protein Data Bank; SARS-CoV, severe acute respiratory syndrome coronavirus; *T*_m_, midpoint transition temperature.

Although Ptal-N*01:01 can bind peptides with Leu as the PΩ anchor, the binding peptides can also possess Pro at this position. This is uncommon in the peptides bound by MHC I molecules from other mammals. The structures of Ptal-N*01:01 with the 9-mer peptides HeV2 (DYINTNVLP) and H17N10-NP (DFEKEGYSL) showed that both of the peptides adopt similar overall conformations as the HLA-A*0201–presented 9-mer peptide GL9 (GILGFVFTL) with a Leu at the PΩ site (Fig 6A). However, to the best of our knowledge, no peptide motif with a Pro at the PΩ position has been reported for HLA-A*0201. Detailed comparative analysis of the F pockets of Ptal-N*01:01 and HLA-A*0201 revealed that the F pocket of Ptal-N*01:01 is shallow but with a wide opening (Fig 6B–6E). Corresponding to Asp^77^ and Tyr^116^ of HLA-A*0201, Ptal-N*01:01 possesses smaller residues Gly^80^ and Leu^119^, respectively. The Ptal-N*01:01–specific Gly^80^ in Ptal-N*01:01 is small, which provides more space to accommodate the pyrrolidine ring of PΩ-Pro. In addition, although both HLA-A*0201 and Ptal-N*01:01 have a similar Lys residue in their corresponding positions (146 for HLA-A*0201 and 149 for Ptal-N*01:01), the conformation of the Lys^149^ of Ptal-N*01:01 is shifted to the C terminus of the PBG, which leaves more space for the open mouth of the F pocket in Ptal-N*01:01.

To verify the allele-specific preference of Ptal-N*01:01 for peptides with Pro at the PΩ site, we examined the binding ability of HLA-A*0201 to a mutated GL9 peptide, GL9-L9P, with a Pro at P9 (Fig 6F and 6G). We found GL9-L9P has a weaker capacity to help the HLA-A*0201 refold, and the generated heterotrimer complex has lower midpoint transition temperature (*T*_m_) (38.1°C) compared with the wild-type peptide GL9 (55.8°C) with a Leu at PΩ. Thus, although Pro at PΩ may also act as a suboptimal anchor for HLA-A*0201, Ptal-N*01:01 uses Pro as one of its optimal PΩ anchors.

The analysis of the 20 different component amino acids for the proteins from the MERS-CoV and SARS-CoV indicated that Pro possesses a relatively low mutation rate compared with the other amino acids (Fig 6H). The mutation rate of Pro is only higher than Trp and Gly, which are the largest and smallest residues, respectively. It indicates that, as special residues Trp and Gly, the residue Pro may also keep the natural conformation and function of viral proteins. In other words, structural constraints favoring conservation of Pro in certain positions of proteins may operate to preserve viral protein conformation and function. Thus, its mutation rate is restricted. The selection of Pro as the anchor residue of bat MHC I Ptal-N*01:01–presented peptides may restrict the viral mutation pushed by T-cell immunity and accelerate virus clearance.

### The deep B pocket of bat MHC I has a different orientation

**Fig 7:**
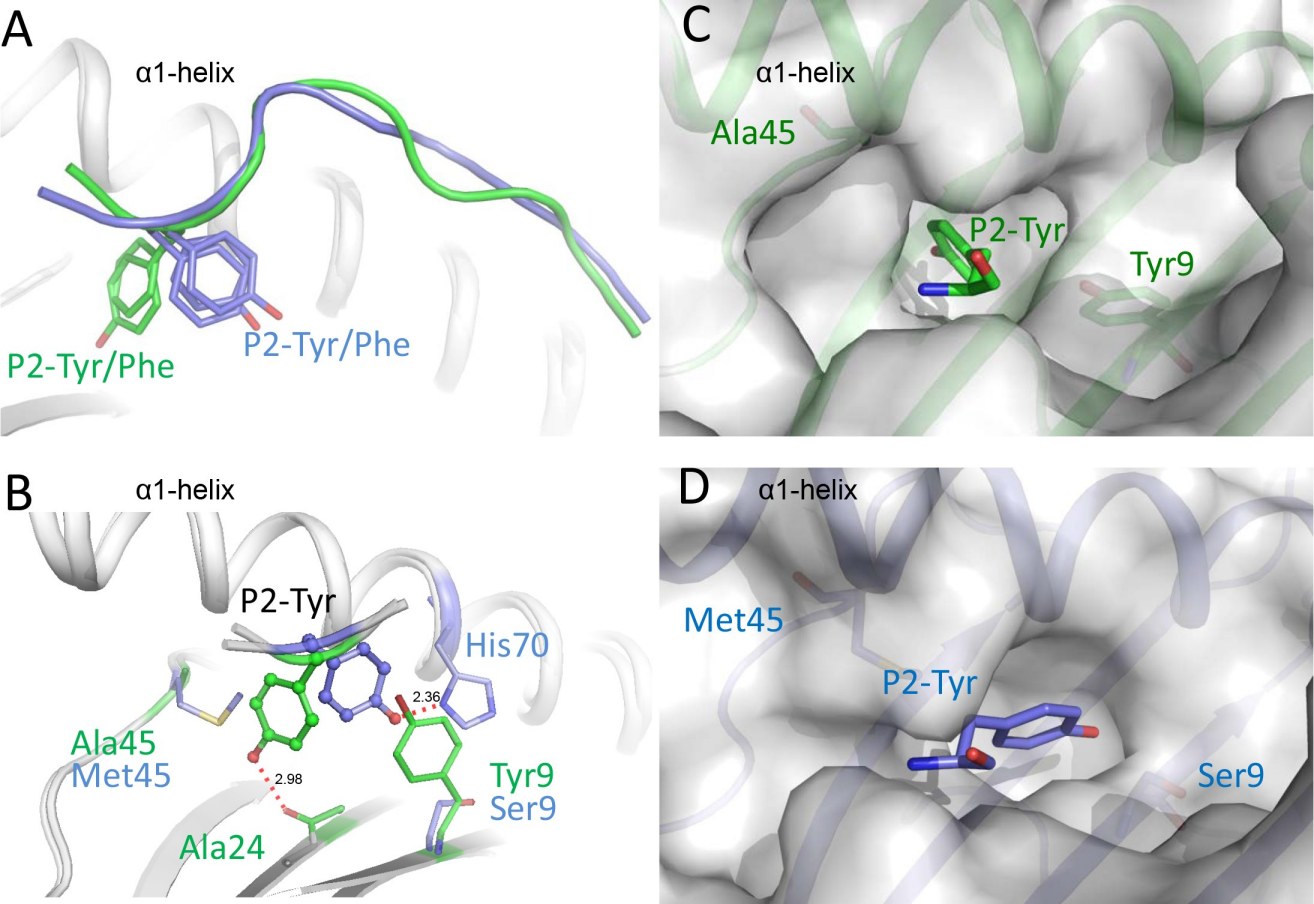
The B pocket of bat MHC I with an uncommon orientation. (A) Structural superimposing of Ptal-N*01:01 with HLA-A*2402 and H-2K^d^. The HLA-A*2402– (PBD code: 3I6L and 5WXD) and H-2K^d^–presented peptides (PBD code: 5GR7) have a P2-Tyr or -Phe (blue sticks) pointing to the C terminus of the PBG, while the P2-Tyr or -Phe (green sticks) of Ptal-N*01:01–binding peptides (HeV1 and HeV2) swing to the N terminus of the PBGs. (B) Different conformations of P2 anchors (shown in sticks and spheres) of Ptal-N*01:01/HeV2 and HLA-A*2402. The major residues of the B pockets of Ptal-N*01:01/HeV2 and HLA-A*2402 (PBD code: 5WXD) are shown as green and blue sticks, respectively. The hydrogen bonds are shown as red dashed lines. (C) The B pocket of Ptal-N*01:01/HeV2 is shown as a semitransparent surface, with Tyr^9^ and Ala^45^ of Ptal-N*01:01 shown as sticks under the surface. Ptal-N*01:01 heavy chain is shown as green cartoons under the semitransparent surface. (D) The B pocket of HLA-A*2402 (PBD code: 5WXD) is shown as a semitransparent surface. The residues Ser^9^ and Met^45^ of HLA-A*2402 are shown as sticks under the surfaces. The different orientation of the P2 residue (blue sticks) of HLA-A*2402–presented peptide is compared with the corresponding residue of Ptal-N*01:01–presented peptide HeV2 in panel C. HeV, Hendra virus; MHC, major histocompatibility complex; PBD, peptide binding groove; PBG, peptide binding groove; P2, position 2.

The P2 positions of Ptal-N*01:01–binding peptides are predominantly aromatic amino acids such as Tyr and Phe as the anchor, which is common in human HLA-A*2402 or mouse H-2K^d^. However, when we superimposed the structures of Ptal-N*01:01 onto the previously determined structures of HLA-A*2402 and H-2K^d^, we found that the P2 anchors of Ptal-N*01:01 protrude in a different direction (Fig 7A). The HLA-A*2402– and H-2K^d^–presented peptides have a Tyr or Phe pointing to the C terminus of the PBG, while the Tyr or Phe of Ptal-N*01:01–binding peptides swing toward the N terminus of the PBG (Fig 7C and 7D). Comparison of the amino acids lining the B pockets of these MHC I molecules from different mammals demonstrated that Ptal-N*01:01 possesses a Tyr^9^ (compared with the small residues Ser^9^ in HLA-A*2401 or Val^9^ in H-2K^d^), which takes up space and can push a P2-Tyr or -Phe to the other direction (Fig 7B). Furthermore, compared with the residues Met^45^ in HLA-A*2402 or Phe^45^ in H-2K^d^, the Ptal-N*01:01–specific Ala^45^ leaves a large space to accommodate the P2-Tyr or -Phe. Indeed, detailed analyses showed that the P2-Tyr of Ptal-N*01:01–binding peptides form hydrogen bonds directly with the main chain of the β-sheet on the floor of the PBG. In contrast, the P2-Tyr of HLA-A*2402–restricted peptides bind to the side chain of His70 on the α1-helix (Fig 7B). Sequence superimposition of the bat MHC I with other mammal MHC Is indicated that Ala^45^ is prevalent (41%) among bat MHC I molecules but is never seen in human and mouse MHC I (S2A Fig).

### Ptal-N*01:01 does not bind to long peptides in an N-terminal extended manner

Previously, it was indicated that Ptal-N*01:01 has a special preference for the binding of long peptides, together with the common 8–10-mer peptides in other mammal MHC I molecules. To further elucidate whether the 3-aa insertion of Ptal-N*01:01 has an impact on the preference for long peptides through an N-terminal extension manner, we synthesized 20 long peptides (11-mer to 15-mer) that were previously eluted from Ptal-N*01:01–expressing cells (S4 Table) [30]. None of these peptides could facilitate Ptal-N*01:01 refolding in vitro (S6A Fig). Considering that these 20 peptides may not have the typical motif of Ptal-N*01:01–binding peptides, we also synthesized naturally N-terminally extended peptides based on the Ptal-N*01:01–binding peptides from HeV1, MERS-CoV-S7, and EBOV-NP1 (S5 Table). Although these three peptides have a typical motif of Ptal-N*01:01–binding peptides, the N-terminal extension led to a failure in peptide binding (S6B Fig and S5 Table). These data indicate that the 3-aa insertion into Ptal-N*01:01 leads to a more restrictive binding peptide selection for Ptal-N*01:01 but not an extension to longer peptides via the N terminus.

## Discussion

The identification of bats as natural reservoirs of several highly pathogenic viruses that impact human and animal health and the fact that these viruses are harmless to bats have resulted in an increasing interest in the investigation of the specificities of the bat immune system. Herein, we screened and identified a series of bat MHC I Ptal-N*01:01–binding peptides derived from four different bat-borne viruses: HeV, EBOV, MERS-CoV, and H17N10. The subsequent determination of the structures of Ptal-N*01:01 complexed with peptides from these viruses revealed unusual peptide presentation features of bat MHC I. Interestingly, this uncommon feature of pocket A of bat MHC I may be shared by the MHC Is from different marsupials. In addition, as the traditional primary anchoring positions for peptides, the B and F pockets of Ptal-N*01:01 also display unconventional conformations that contribute to the distinct peptide presentation and special peptide motif compared with other higher mammals.

The sequence combination of the 3-aa insertion at the N terminus of the α1-helix and the charge matching residues at positions 59/65 enable an unusually tight anchoring of the P1-Asp in pocket A of Ptal-N*01:01. But more significantly, this insertion site is located at a position called 3_10_ helix (residues 49–53); newly synthesized MHC I molecules complexed with β_2_m are poised in the endoplasmic reticulum in a peptide-receptive (PR) form, ready to bind and be stabilized in the mature peptide-loaded (PL) form by peptides destined for display at the cell surface. The movement of a hinged unit containing a conserved 3_10_ helix promotes the PR transition state to a PL mature molecule [31]. Chaperone-mediated loading of high-affinity peptides onto MHC I is a key step in the MHC I antigen presentation pathway. TAP binding protein (TAPBPR; related) remodels the peptide-binding groove of MHC I, resulting in the release of low-affinity peptide [32]. In the absence of TAPBPR, Y84 (Y87 in Ptal-N*01:01) plays a role in closing of the α2-1-helix “latch” by associating with the C terminus of bound peptides in the F pocket, the release of nonoptimal peptides induced by TAPBPR [33]. However, the hydrogen bond network of the A pocket could stabilize peptides with P1-Asp during peptide processing and exchange and high-affinity peptides are guided by initial contacts spanning both the A- and F pockets to form a prolonged interaction within the groove, resulting in a closure of the α2–1 helix latch, which triggers TAPBPR release from the peptide/MHC I complex.

Being highly polymorphic, subtle substitutions or insertions in the PBGs of MHC I molecules may dramatically affect the binding peptide pool and also the peptide presentation to T cells [34,35]. Comparative genomic and transcriptomic analysis demonstrated that a series of bat MHC I molecules have an insertion in the N terminus of the PBG compared with other higher mammals [29]. Herein, our structural study visually shows that the 3-aa insertion in the bat MHC I leads to an extension of the α1-helix. This conformational change causes the protrusion of the bat-specific residue Asp^59^ into the A pocket of the PBG, which forms a hydrogen bond network with the Arg^65^ and the peptide P1-Asp. Study of the peptide repertoire of key human and mouse MHC I alleles demonstrates that anchor Asp is disfavored at the P1 position of the peptides [36]. In contrast, recently reported data on the peptide elution from bat MHC I molecules [30], together with viral peptide screening results in this study, demonstrate that bat MHC I Ptal-N*01:01 prefers peptides with a P1-Asp. Either depletion of the three inserted residues (Met^52^Asp^53^Leu^54^) or substitution of the P1-Asp with Ala impaired the MHC/peptide binding. Indeed, the electrostatic nature of the contacts between Asp^59^, Arg^65^, and P1-Asp and the fact that it is involved in solvent-exposed elements in pocket A of the MHC complex suggest that the P1-Asp acts as a “surface anchor residue.” This surface anchor residue was also previously defined in a phosphopeptide-MHC complex [37] in which the solvent-exposed P4 phosphate moiety can enhance the stability of the peptide-MHC association. The additional surface anchor residue for the bat MHC I binding peptides may have at least two advantages for antiviral T-cell immunity. First, the peptides tightly bind to Ptal-N*01:01 and present a special peptide-MHC landscape at the N terminus of the peptides for the T-cell recognition. Second, negatively charged residues such as Asp/Glu in the peptides may also act as key residues in the original viral proteins for virus replication, which will have lower mutation rates to escape T-cell recognition. In addition, the insertion of Met^52^Asp^53^Leu^54^ also leads to a special exposed landscape of the α1-helix of Ptal-N*01:01. Thus, heterozygous bats with MHC I alleles of both 3-aa insertion and no insertion may possess a T-cell repertoire with broader diversity, which need more work to investigate.

Bats are one of the most ancient extant lineages of eutherian mammals, believed to be located in distinct mammalian lineages different from marsupials and other higher eutherians [29]. The evolution of the MHC I gene family is closely tied to the evolution of the vertebrates genome [38]. However, the 3-aa insertion and the charge-matching residues at positions 59/65 of MHC Is are also prevalent among different marsupials: opossum, koala, tammar wallaby, and Tasmanian devil. This may be a phenomenon of convergent evolution under the pressure of related pathogens. To comparatively investigate the peptide presentation of MHC I from marsupials, we have synthesized a marsupial MHC I gene (Trvu-UB*01), which has similar characteristics to 3-aa insertion and charge-matching residues at positions 59/65 to Ptal-N*01:01, and also the small residue G at position 80 (S7A Fig). However, the preference of the Trvu-UB*01 protein for peptides may not be similar to that of Ptal-N*01:01 (S7B Fig). This result indicates that although some alleles of MHC I from the marsupials possess the same key residues in the PBG of Ptal-N*01:01, the preferred peptide motifs are only partially similar between them (Pro as PΩ), which may reflect the contribution of some other adjacent residues. Thus, the detailed peptide motifs and the presentation features of MHC Is from these lower mammals still require further laboratory investigation. Meanwhile, whether the 5-aa insertion in the bat MHC I impacts the peptide presentation in the same manner as 3-aa insertion or with a new molecular mechanism needs more structural studies. In this context, it is also worth mentioning that we also tried two additional online MHC-peptide binding predicting servers, NetMHCpan and Rosetta FlexPepDock, to verify the peptide-binding experiments in the study. However, neither prediction server was able to match the experimental results. This may indicate that current MHC binding peptide predictions were not suitable for non-mouse and nonhuman mammals such as bats, which may have a different manner of peptide binding.

Like the human and murine MHC I [2,39], Ptal-N*01:01 has B and F pockets in the PBG to accommodate the primary anchors of the binding peptides, but uncommon conformational features of Ptal-N*01:01–loaded B and F pockets were identified through our structural investigations. The B pocket of Ptal-N*01:01, featuring the bat-specific residue Ala^45^, has a novel position in the PBG for the P2 anchor. In this position, the P2-Tyr of the peptides form hydrogen bonds directly with the main chain of the β-sheet on the floor of the PBG of Ptal-N*01:01, which leads to a stable anchoring of the P2-Tyr. Meanwhile, Ptal-N*01:01, with a relatively unique Gly at position 80, has an unusually shallow F pocket with a wide entrance, like a large bowl, which can accommodate the uncommon PΩ-Pro anchor. To the best of our knowledge, although the PΩ-Pro anchor is not observed in the previously reported peptides from mammalian MHC I, the precedents for the P2-Pro anchor have been reported in the context of murine H-2L^d^ and human B*3501 and B*5301 [40–42]. Requirements for Pro in the P3 position as an anchor residue are also observed in the murine H-2D^d^- and macaque Mamu-A*01–restricted epitopes [43,44]. A previous study indicates that the antiviral effect of T cells is sufficiently strong to force the virus to adopt a relatively unfavorable mutation, which reduces viral replication [45]. The proline anchor may be a result of special antigen processing in bats. For humans, most products of ornithine decarboxylase degraded in vitro by the 26 S ATP-dependent proteasome, which contained one or two Pro residues, implied that the Pro residue has a role in the escape from random cleavage by proteasomes [46]. In addition, Pro residue(s) within epitopic sequences presumably contribute to efficient production of MHC class I ligands through prevention of their random cleavage by proteasomes [47]. Thus, the peptides with proline as a C terminus are still seldom in human and other common mammals. However, the current research on the proteasome of bats is still blank. Our data also showed that among the 20 different component amino acids for the proteins from the bat-related viruses, the residue Pro possesses a relatively low mutation rate compared with the other amino acids (Fig 6H). Pro, with a unique conformation, may act as a key residue for the structure and function of viral proteins, and thus its mutation rate is low. Therefore, the usage of Pro as the PΩ anchor of the T-cell epitopes in Ptal-N*01:01–carrying bats may also restrict the formation of escape mutations. However, based on the currently limited amount of bat MHC I sequences available, Gly^80^ in the F pocket does not seem prevalent in bat MHC I. More sequencing of bat genomes and especially MHC I genes are needed to verify whether the accommodation of PΩ-Pro as a peptide anchor is common in bat MHC I or a specific feature of Ptal-N*01:01.

In conclusion, through a series of structural and functional investigation, we demonstrated several novel features of bat MHC class I molecules presenting virus-derived peptides. Our results provide new insight into the adaptive immune system of bats, which may contribute to the unique virus–host interactions in these important mammals. Due to the high containment nature of the viruses and the difficulty in conducting live bat infection studies, our current study lacks in vivo functional characterization, which we hope to conduct in the future with international collaborations.

## Materials and methods

### Sequence retrieval and analyses

The sequences of 56 MHC class I genes (including predicted genes) from bats were retrieved from the NCBI database (S1 Table). Higher mammal MHC I heavy chain sequences were retrieved from the Immuno Polymorphism Database (IPD) (www.ebi.ac.uk/ipd/mhc) and the UniProt database (www.uniprot.org). Previously deposited marsupial (opossum, tammar wallaby, koala, Tasmanian devil) and platypus MHC I transcripts were included in these analyses (S1 Table). Sequence alignments were generated with ClustalX [48] and ESPript [49]. Similarities were calculated using DNAMAN (https://www.lynnon.com/).

The proteomes of 1,000 MERS-CoV genomes and 1,000 SARS-CoV genomes were retrieved from GenBank, respectively. After sequence alignment with MAFFT, the dominant amino acid for each site was elected as a reference sequence. The mutation frequency = the number of overall mutations for each amino acid/(the number of occurrences of the amino acid in the reference sequence×total number of sequences).

### Peptide synthesis and preparation of expression constructs

To screen potential peptides for binding to Ptal-N*01:01, the proteomes of the bat-related viruses EBOV (NP: GenBank no. AF054908.1; GP: GenBank no. AKG65250.1), MERS-CoV (GenBank no. AXN92228.1), H17N10 influenza-like virus (A/little yellow-shouldered bat/Guatemala/060/2010(H17N10)), and H18N11 influenza-like virus (A/flat-faced bat/Peru/033/2010(H18N11)) were utilized to predict the candidate peptides. The candidate peptides were predicted and selected according to the recently reported motif, by which the two Ptal-N*01:01–binding peptides, HeV1 and HeV2, derived from HeV were also synthesized (S2 Table) [30]. The potential binding scores of the selected peptides were also predicted through the online NetMHCpan 4.0 server (http://www.cbs.dtu.dk/services/NetMHCpan/) [50] and Rosetta FlexPepDock, which is based on structure modeling [51,52], so that we prefer choose peptides that conform to the motif of Ptal-N*01:01 [30]. The peptide purity was determined to be >95% by analytical HPLC and mass spectrometry. The peptides were stored at −80°C as freeze-dried powders and were dissolved in DMSO before use.

The cDNAs for the heavy chain of *P*. *alecto* MHC I Ptal-N*01:01 (GenBank no. KT987929) [30] and bat β_2_m (GenBank no. XP_006920478.1) were synthesized (Genewiz, Beijing, China). Ptal-N*01:01 sequence was deposited to GenBank by Wynne and colleagues, and Ptal-N*01:01–binding peptides HeV1 and HeV2 were identified in their study [30]. Although Ng and colleagues reported the first Ptal-N*01:01 [29], the sequence is not available online. To investigate the function of Met^52^Asp^53^Leu^54^ in Ptal-N*01:01, a mutant termed Ptal-N*01:01(-3aa) with a deletion of these three amino acids was constructed. The amplified products expressing the extracellular domain (residues 1–277) of Ptal-N*01:01 and bat β_2_m (residues 1–98) were cloned into a pET28a vector (Novagen). The expression plasmid for human β_2_m (residues 1–99) was previously constructed in our laboratory [53].

### Refolding and purification of bat class I complexes

Renaturation and purification of Ptal-N*01:01 assembled with peptides were performed as previously described [54,55]. Generally, bat MHC I Ptal-N*01:01 heavy chain and bat β_2_m were overexpressed as inclusion bodies in the BL21(DE3) strain of *Escherichia coli*, and the purified inclusion bodies of the proteins were solubilized in 6 M guanidine-HCl buffer with a concentration of 30 mg/mL. Then, injection and dilution of MHC heavy chain, β_2_m, and peptide occurred at a molar ratio of 1:1:3 in refolding buffer (100 mM Tris-HCl [pH 8], 2 mM EDTA, 400 mM L-Arg, 0.5 mM oxidized glutathione, and 5 mM reduced glutathione) [34]. After 24 hours for protein refolding, the Ptal-N*01:01 complexes were concentrated and exchanged into a buffer of 20 mM Tris-HCl (pH 8) and 50 mM NaCl and then purified using a Superdex 200 16/60 HiLoad (GE Healthcare, Beijing, China) size-exclusion column.

### Crystallization, data collection, and processing

Crystallization was performed using the sitting drop vapor diffusion technique. The Ptal-N*01:01/peptide complexes were screened through Crystal Screen kit I/II, Index Screen kit, PEGIon kit I/II, and the PEGRx kit (Hampton Research). Plates were incubated at 291 K and 277 K and assessed for crystal growth after 1–2 weeks. Ptal-N*01:01/HeV1 crystals were observed in 0.2 M NaCl, 0.1 M Bis-Tris (pH 5.5), and 25% (w/v) polyethylene glycol 3,350 at a concentration of 7.5 mg/mL. Ptal-N*01:01/HeV1(human β_2_m) crystals were observed in 0.1 M HEPES, pH 7.0, 2% w/v polyethylene glycol 3,350. Monomer bat β_2_m were grown in 0.1 M BIS-TRIS, pH 6.5, 8% w/v polyethylene glycol monomethyl ether 5,000. Single crystals of Ptal-N*01:01/HeV2 were grown in 0.075 M HEPES (pH 7.5), 15% (w/v) polyethylene glycol 10,000, and 25% (v/v) glycerol at a protein concentration of 10 mg/mL. Single crystals of Ptal-N*01:01/EBOV-NP1 were grown in 0.1 M succinic acid (pH 7.0) and 15% (w/v) polyethylene glycol 3,350. Single crystals of Ptal-N*01:01/EBOV-NP2 were grown in 0.2 M ammonium acetate, 0.1 M Tris (pH 8.0), and 16% (w/v) polyethylene glycol 10,000. Single crystals of Ptal-N*01:01(-3aa)/HeV1 were grown in 0.2 M sodium formate and 20% (w/v) polyethylene glycol 3,350. Single crystals of Ptal-N*01:01/H17N10-NP were grown in 0.075 M HEPES (pH 7.5), 15% (w/v) polyethylene glycol 10,000, and 25% (v/v) glycerol. Single crystals of Ptal-N*01:01/MERS-CoV-S3 were grown in 0.2 M sodium acetate trihydrate and 20% (w/v) polyethylene glycol 3,350. For cryoprotection, crystals were transferred to reservoir solutions containing 20% glycerol and then flash-cooled in a stream of gaseous nitrogen at 100 K. X-ray diffraction data were collected at beamline BL19U of the Shanghai Synchrotron Radiation Facility. The data collection statistics are shown in Table 1.

### Structure determination and analyses

The collected intensities were subsequently processed and scaled using the Denzo program and the HKL2000 software package (HKL Research). The structures were determined using molecular replacement with the program Phaser MR in CCP4 [56]. The model used was the structure coordinates with Protein Data Bank (PDB) code 5F1I [35], and restrained refinement was performed using REFMAC5 from CCP4. Extensive model building was performed by hand using COOT [57]. The stereochemical quality of the final model was assessed with the program REFINE in Phenix or CCP4 (Table 1). Structure-related figures were generated using PyMOL (http://www.pymol.org/) and COOT.

### Determination of protein thermostability using CD spectroscopy

The thermostabilities of Ptal-N*01:01 with two group key peptides were tested by CD spectroscopy. All complexes were refolded, purified, and measured at 0.2 mg/mL in a solution of 20 mM Tris (pH 8) and 50 mM NaCl. CD spectra at 218 nm were measured on a Chirascan spectrometer (Applied Photophysics) using a thermostatically controlled cuvette at temperature intervals of 0.2°C at an ascending rate of 1°C/minute between 20 and 90°C. The unfolded fraction (%) is expressed as (θ−θ_a_)/(θ_a_−θ_b_), where θ_a_ and θ_b_ are the mean residue ellipticity values in the fully folded and fully unfolded states, respectively. The denaturation curves were generated by nonlinear fitting with OriginPro 8.0 (OriginLab) [58]. The *T*_m_ was calculated by fitting data to the denaturation curves and using inflection-determining derivatives.

## Supporting information

S1 Fig Structure-based sequence alignment of Ptal-N*01:01 and other bat MHC I molecules. Coils indicate α-helices, and black arrows indicate β-strands. Residues highlighted in red are completely conserved, and residues in blue boxes are highly (80%) conserved, with consensus amino acids in red. Residues forming the B pocket are marked with a yellow background and the F pocket with blue. The key residues in the pocket are marked with red five-pointed stars. Special insertion positions in Ptal-N*01:01 are marked with red arrows. The sequence alignment was generated with MEGA7, ClustalX, and ESPript. MHC, major histocompatibility complex.
(TIF)

S2 Fig Structure-based sequence alignment of representative MHC I molecules from bats, marsupials, and higher mammals. (A) Coils indicate α-helices, and black arrows indicate β-strands. Residues highlighted in red are completely conserved, and residues in blue boxes are highly (80%) conserved, with consensus amino acids in red. Special insertion positions in Ptal-N*01:01 are marked with red arrows. The sequence alignment was generated with MEGA7, ClustalX, and ESPript. (B) Structure-based sequence alignment of β_2_m derived from different mammals. Residues binding to α1α2 domains of the Ptal-N*01:01 heavy chain were labeled by black triangles. Residues binding to α3 domains of the Ptal-N*01:01 heavy chain were labeled by red triangles. The conserved residues between bat β_2_m and human β_2_m were labeled by filled triangles, and the variable residues between bat β_2_m and human β_2_m were labeled by hollow triangles. β_2_m, β_2_-microglobulin; MHC, major histocompatibility complex.
(TIF)

S3 Fig The conformations and electron density maps of Ptal-N*01:01–presented peptides. The authentic conformations of HeV1 (A), HeV2 (B), H17N10-NP (C), EBOV-NP1 (D), EBOV-NP2 (E), and MERS-S3 (F) presented by Ptal-N*01:01 are shown through the 2Fo-Fc electron density maps contoured at a contour of 1.0σ viewed in profile through the α2-helix. The electron density maps were constructed from model phases, omitting the peptides. The peptides are displayed as sticks in different colors. The hypothetical P3–P8 residues of the peptide MERS-S3 with poor electron densities were denoted as dashed orange lines. EBOV, Ebola virus; HeV, Hendra virus; MERS, Middle East respiratory syndrome.
(TIF)

S4 Fig The surface profile of pocket A of Ptal-N*01:01 compared with the MHC I molecules from other vertebrates. The surface profile of the A pocket of bat MHC I Ptal-N*01:01 was compared with the A pockets of MHC I from other vertebrates, including human HLA-A*0201, HLA-A*2402, murine H-2K^d^, chicken BF2*04, and BF2*21. The yellow arrows indicate the vacant edge of the A pockets of the MHC I from vertebrates other than bat. All of the pockets are shown as semitransparent electron density maps, under which the Asp^59^ and Arg^65^ of Ptal-N*01:01 and the corresponding residues in other MHC I were shown as sticks. The P1 anchors in different MHC I were represented with gray sticks and spheres. MHC, major histocompatibility complex; P1, position 1.
(TIF)

S5 Fig The consistent hydrogen bond network in the A pockets of six Ptal-N*01:01 structures. The A pockets of bat MHC I Ptal-N*01:01 structures complexed with peptides HeV1 (A), HeV2 (B), H17N10-NP (C), EBOV-NP1 (D), EBOV-NP2 (E), and MERS-CoV-S3 (F) from different viruses. Residues Asp^59^ and Arg^65^ in the A pocket of Ptal-N*01:01 are shown as sticks, and the P1 anchor Asp of these peptides are represented as sticks and spheres. The hydrogen bonds are denoted in dashed lines. The heavy chains of different Ptal-N*01:01 structures are shown in white cartoon. EBOV, Ebola virus; HeV, Hendra virus; MERS-CoV, Middle East respiratory syndrome coronavirus; MHC, major histocompatibility complex; P1, position 1.
(TIF)

S6 Fig The binding capabilities of long peptides with Ptal-N*01:01. (A) Binding of long peptides (11-mers to 15-mers) to Ptal-N*01:01 elucidated by in vitro refolding. Twenty long peptides (peptide Bat1 to Bat20) that were previously eluted from Ptal-N*01:01–expressing cells were synthesized (S4 Table) [30]. The gray curve is a negative control without any peptide in the refolding reaction. (B) Capability of naturally N-terminally extended peptides HeV1 (DFANTFLP), MERS-CoV-S7 (DFNLTLLEP), and EBOV-NP1 (DFQESADSFL) to renature Ptal-N*01:01. EBOV, Ebola virus; HeV, Hendra virus; MERS-CoV, Middle East respiratory syndrome coronavirus.
(TIF)

S7 Fig The identification of marsupial related virus-derived peptides binding to Trvu-UB*01. (A) Structure-based sequence alignment of Ptal-N*01:01 and Trvu-UB*01. (B-C) The peptide predictions refer to pocket features. The binding of peptides derived from possum nidovirus with Trvu-UB*01 were evaluated by co-refolding; the peptide sequence is listed.
(TIF)

S1 Table Sequence information of bats and typical marsupials. Raw data corresponding to Fig 1.
(DOCX)

S2 Table Characteristics of the virus-peptides used in this study. Raw data corresponding to Fig 2.
(DOCX)

S3 Table Binding assays of Ptal-N*01:01 with HeV1/HeV2 and its mutants. **Peptides’ information corresponding to Fig 4.** HeV, Hendra virus.
(DOCX)

S4 Table Long peptides’ information corresponding to S6 Fig. (DOCX)

S5 Table Mutant peptides’ information corresponding to S6 Fig. (DOCX)

S1 Data Numerical data underlying Figs 1A, 1C, 1D, 4H, 4J, 5I, 5L and 6G. (XLSX)

S2 Data Sequence information and Python script corresponding to Fig 6H. (ZIP)

## Abbreviations

aa: amino acid
APC: antigen presenting cell
β_2_m: β_2_-microglobulin
CD: circular dichroism
EBOV: Ebola virus
HeV: Hendra virus
IFN: interferon
Ig: immunoglobulin
IPD: Immuno Polymorphism Database
MERS-CoV: Middle East respiratory syndrome coronavirus
MHC: major histocompatibility complex
NHP: nonhuman primate
NK: natural killer
PBG: peptide binding groove
PDB: Protein Data Bank
PL: peptide-loaded
PR: peptide-receptive
P1: position 1
RMSD: root mean square deviation
SADS-CoV: swine acute diarrhea syndrome coronavirus
SARS-CoV: severe acute respiratory syndrome coronavirus
STING: stimulator of interferon genes
TAPBPR: TAP binding protein
TCR: T-cell receptor
*T*_m_: midpoint transition temperature
