# Supplementary figures and images for: Peptide presentation by bat MHC class I provides new insight into the antiviral immunity of bats
Source: PLoS Biol. 2019 Sep 9;17(9):e3000436. doi: 10.1371/journal.pbio.3000436 (PMC6752855; doi:10.1371/journal.pbio.3000436)

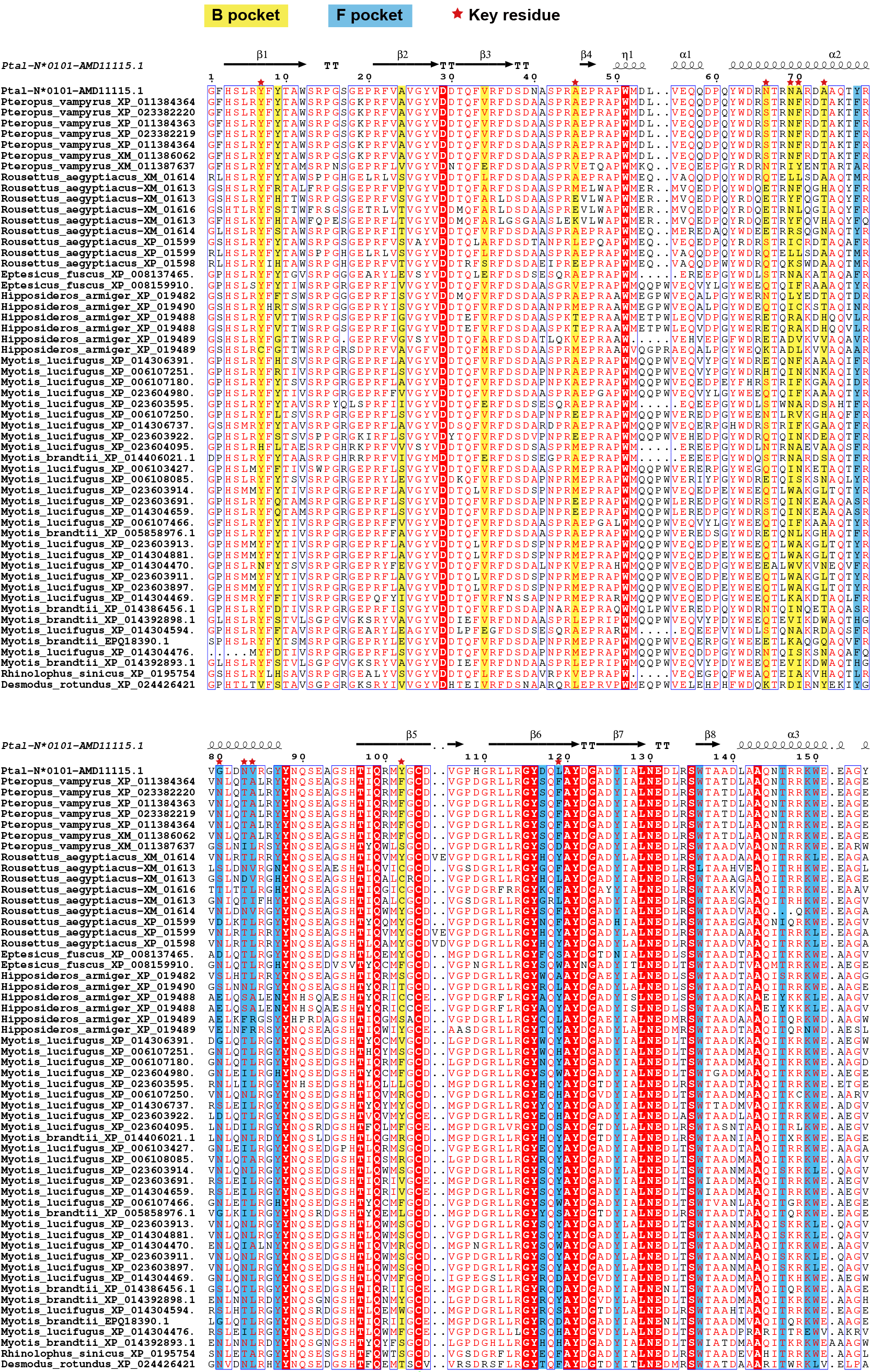

Supplement: S1 Fig — Coils indicate α-helices, and black arrows indicate β-strands. Residues highlighted in red are completely conserved, and residues in blue boxes are highly (80%) conserved, with consensus amino acids in red. Residues forming the B pocket are marked with a yellow background and the F pocket with blue. The key residues in the pocket are marked with red five-pointed stars. Special insertion positions in Ptal-N*01:01 are marked with red arrows. The sequence alignment was generated with MEGA7, ClustalX, and ESPript. MHC, major histocompatibility complex. (TIF) [file pbio.3000436.s001.tif]

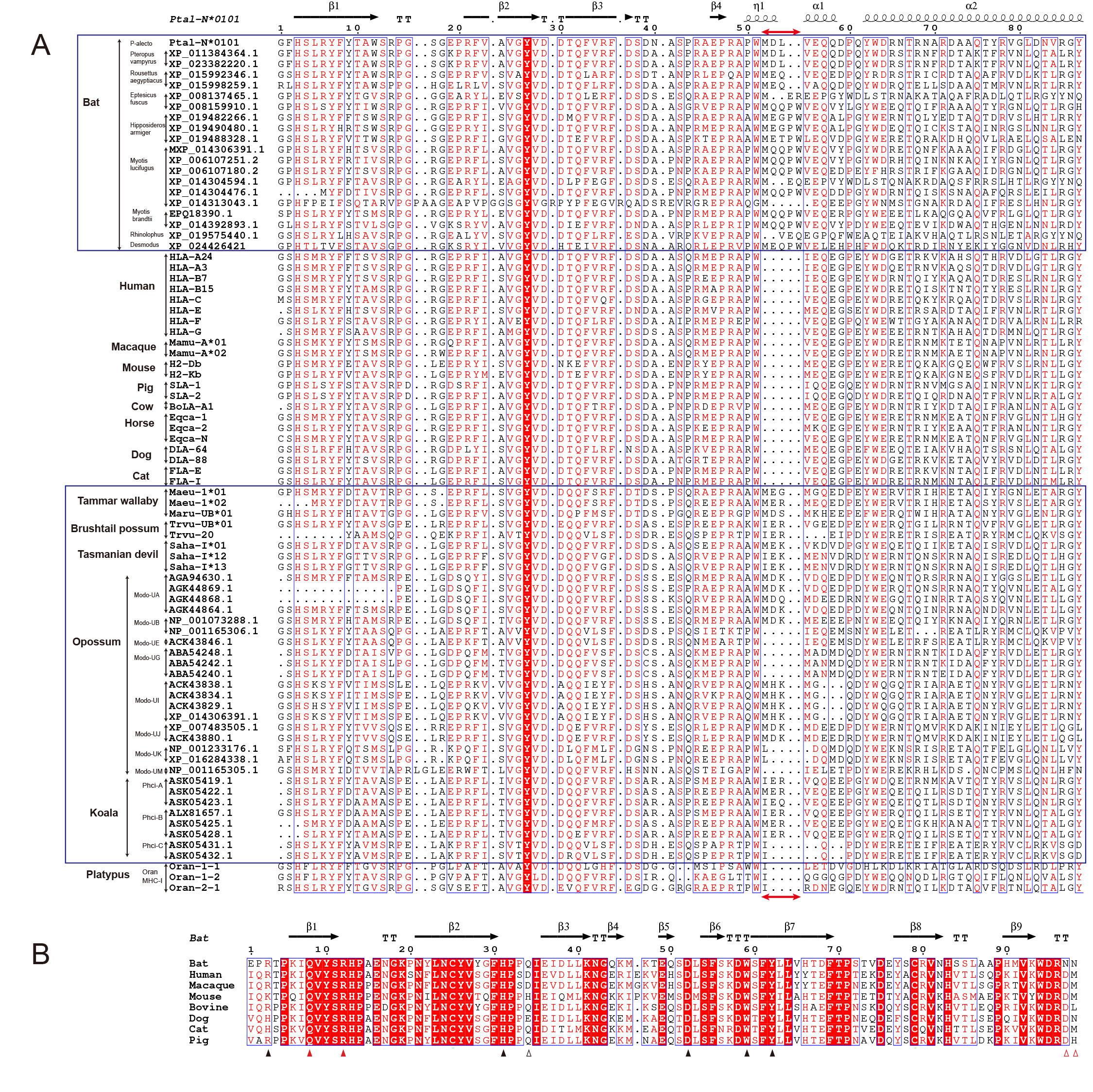

Supplement: S2 Fig — (A) Coils indicate α-helices, and black arrows indicate β-strands. Residues highlighted in red are completely conserved, and residues in blue boxes are highly (80%) conserved, with consensus amino acids in red. Special insertion positions in Ptal-N*01:01 are marked with red arrows. The sequence alignment was generated with MEGA7, ClustalX, and ESPript. (B) Structure-based sequence alignment of β2m derived from different mammals. Residues binding to α1α2 domains of the Ptal-N*01:01 heavy chain were labeled by black triangles. Residues binding to α3 domains of the Ptal-N*01:01 heavy chain were labeled by red triangles. The conserved residues between bat β2m and human β2m were labeled by filled triangles, and the variable residues between bat β2m and human β2m were labeled by hollow triangles. β2m, β2-microglobulin; MHC, major histocompatibility complex. (TIF) [file pbio.3000436.s002.tif]

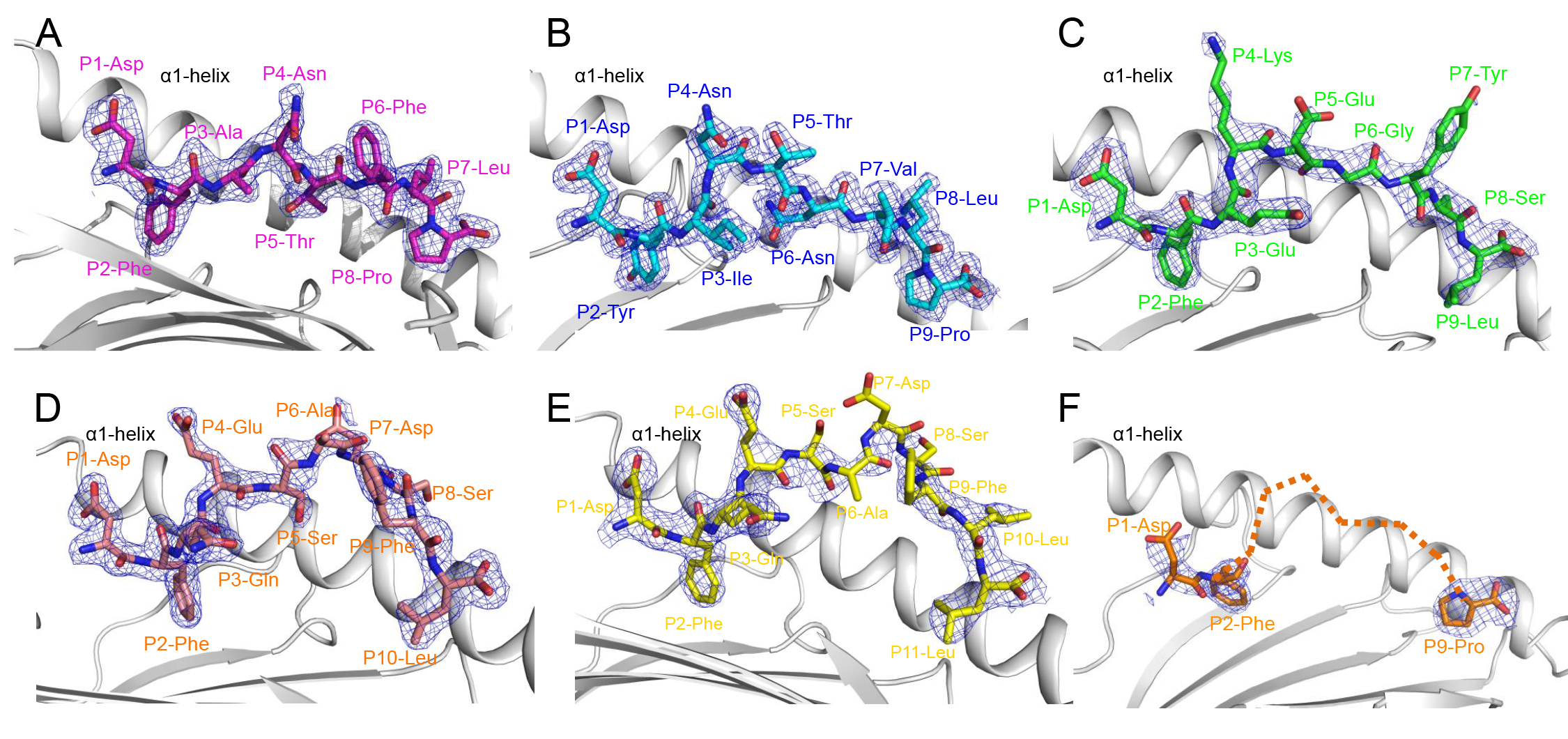

Supplement: S3 Fig — The authentic conformations of HeV1 (A), HeV2 (B), H17N10-NP (C), EBOV-NP1 (D), EBOV-NP2 (E), and MERS-S3 (F) presented by Ptal-N*01:01 are shown through the 2Fo-Fc electron density maps contoured at a contour of 1.0σ viewed in profile through the α2-helix. The electron density maps were constructed from model phases, omitting the peptides. The peptides are displayed as sticks in different colors. The hypothetical P3–P8 residues of the peptide MERS-S3 with poor electron densities were denoted as dashed orange lines. EBOV, Ebola virus; HeV, Hendra virus; MERS, Middle East respiratory syndrome. (TIF) [file pbio.3000436.s003.tif]

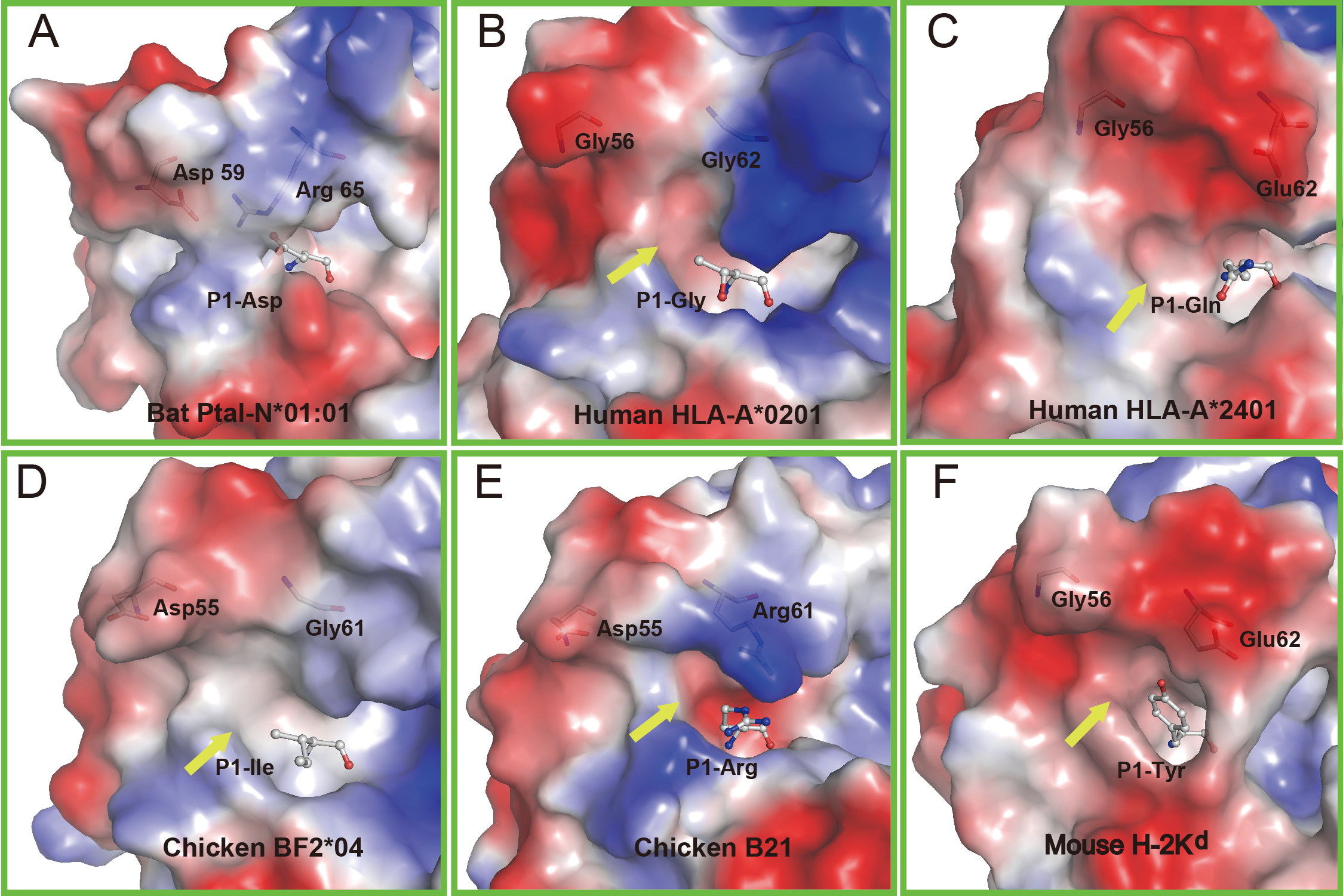

Supplement: S4 Fig — The surface profile of the A pocket of bat MHC I Ptal-N*01:01 was compared with the A pockets of MHC I from other vertebrates, including human HLA-A*0201, HLA-A*2402, murine H-2Kd, chicken BF2*04, and BF2*21. The yellow arrows indicate the vacant edge of the A pockets of the MHC I from vertebrates other than bat. All of the pockets are shown as semitransparent electron density maps, under which the Asp59 and Arg65 of Ptal-N*01:01 and the corresponding residues in other MHC I were shown as sticks. The P1 anchors in different MHC I were represented with gray sticks and spheres. MHC, major histocompatibility complex; P1, position 1. (TIF) [file pbio.3000436.s004.tif]

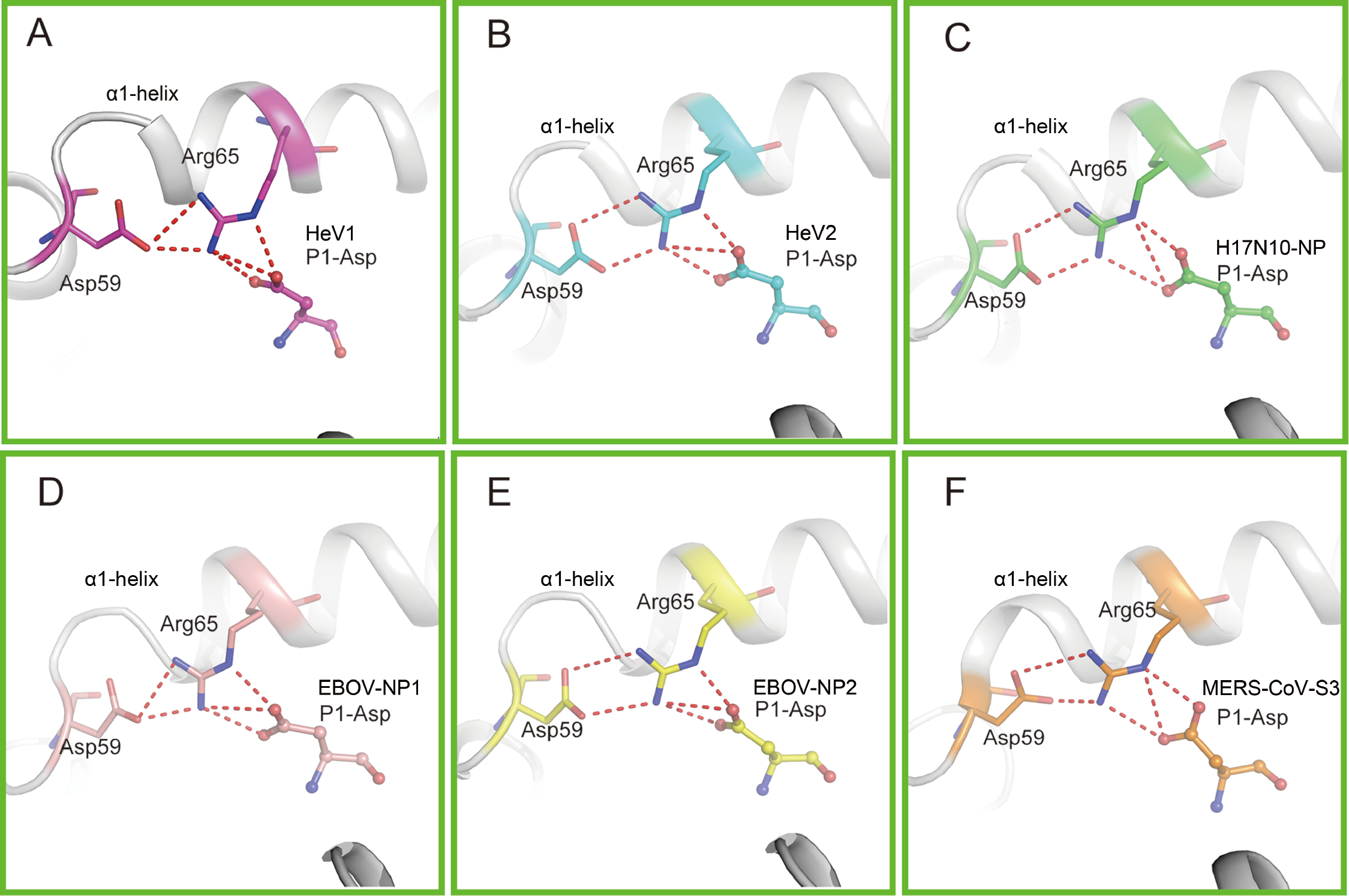

Supplement: S5 Fig — The A pockets of bat MHC I Ptal-N*01:01 structures complexed with peptides HeV1 (A), HeV2 (B), H17N10-NP (C), EBOV-NP1 (D), EBOV-NP2 (E), and MERS-CoV-S3 (F) from different viruses. Residues Asp59 and Arg65 in the A pocket of Ptal-N*01:01 are shown as sticks, and the P1 anchor Asp of these peptides are represented as sticks and spheres. The hydrogen bonds are denoted in dashed lines. The heavy chains of different Ptal-N*01:01 structures are shown in white cartoon. EBOV, Ebola virus; HeV, Hendra virus; MERS-CoV, Middle East respiratory syndrome coronavirus; MHC, major histocompatibility complex; P1, position 1. (TIF) [file pbio.3000436.s005.tif]

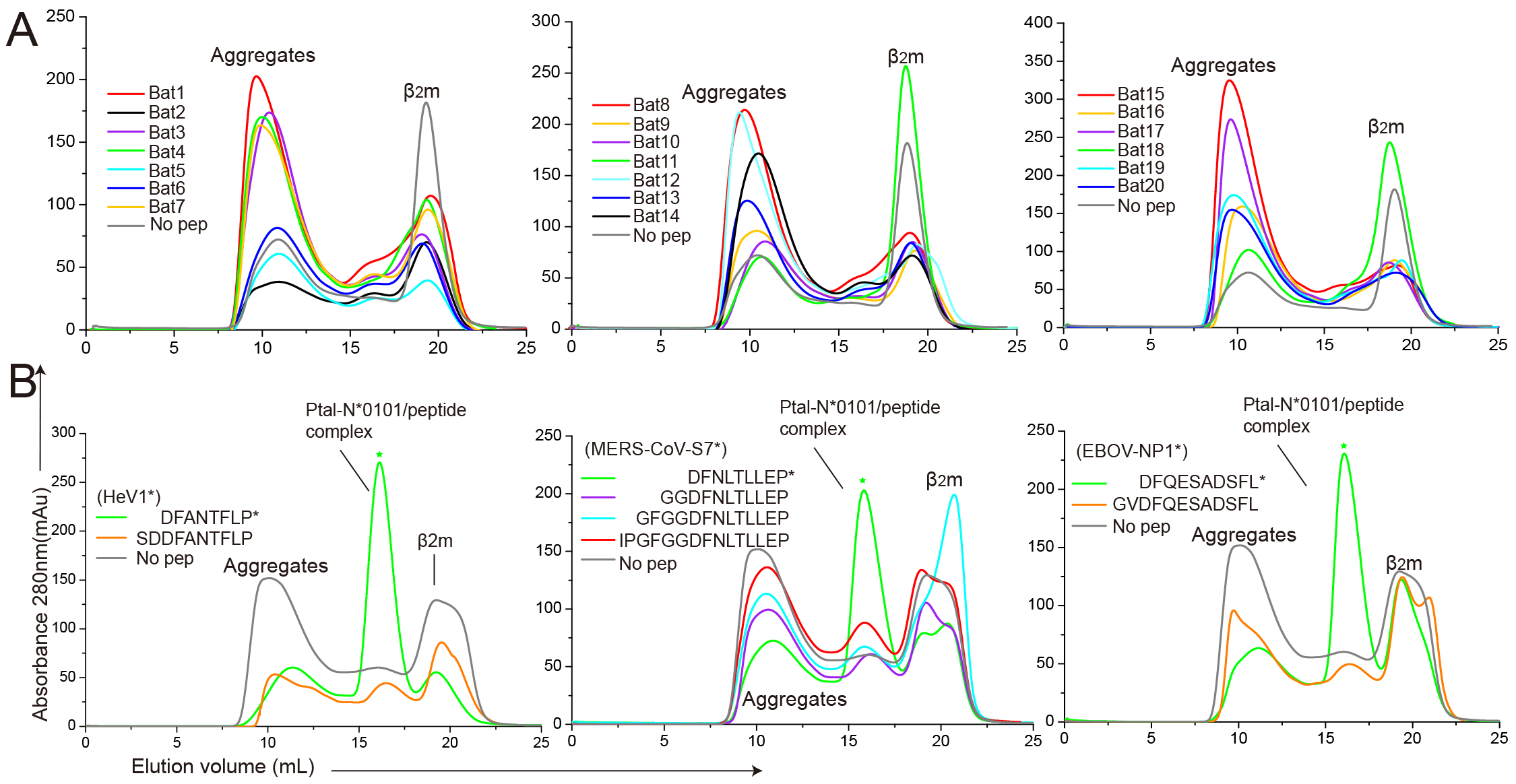

Supplement: S6 Fig — (A) Binding of long peptides (11-mers to 15-mers) to Ptal-N*01:01 elucidated by in vitro refolding. Twenty long peptides (peptide Bat1 to Bat20) that were previously eluted from Ptal-N*01:01–expressing cells were synthesized (S4 Table) [30]. The gray curve is a negative control without any peptide in the refolding reaction. (B) Capability of naturally N-terminally extended peptides HeV1 (DFANTFLP), MERS-CoV-S7 (DFNLTLLEP), and EBOV-NP1 (DFQESADSFL) to renature Ptal-N*01:01. EBOV, Ebola virus; HeV, Hendra virus; MERS-CoV, Middle East respiratory syndrome coronavirus. (TIF) [file pbio.3000436.s006.tif]

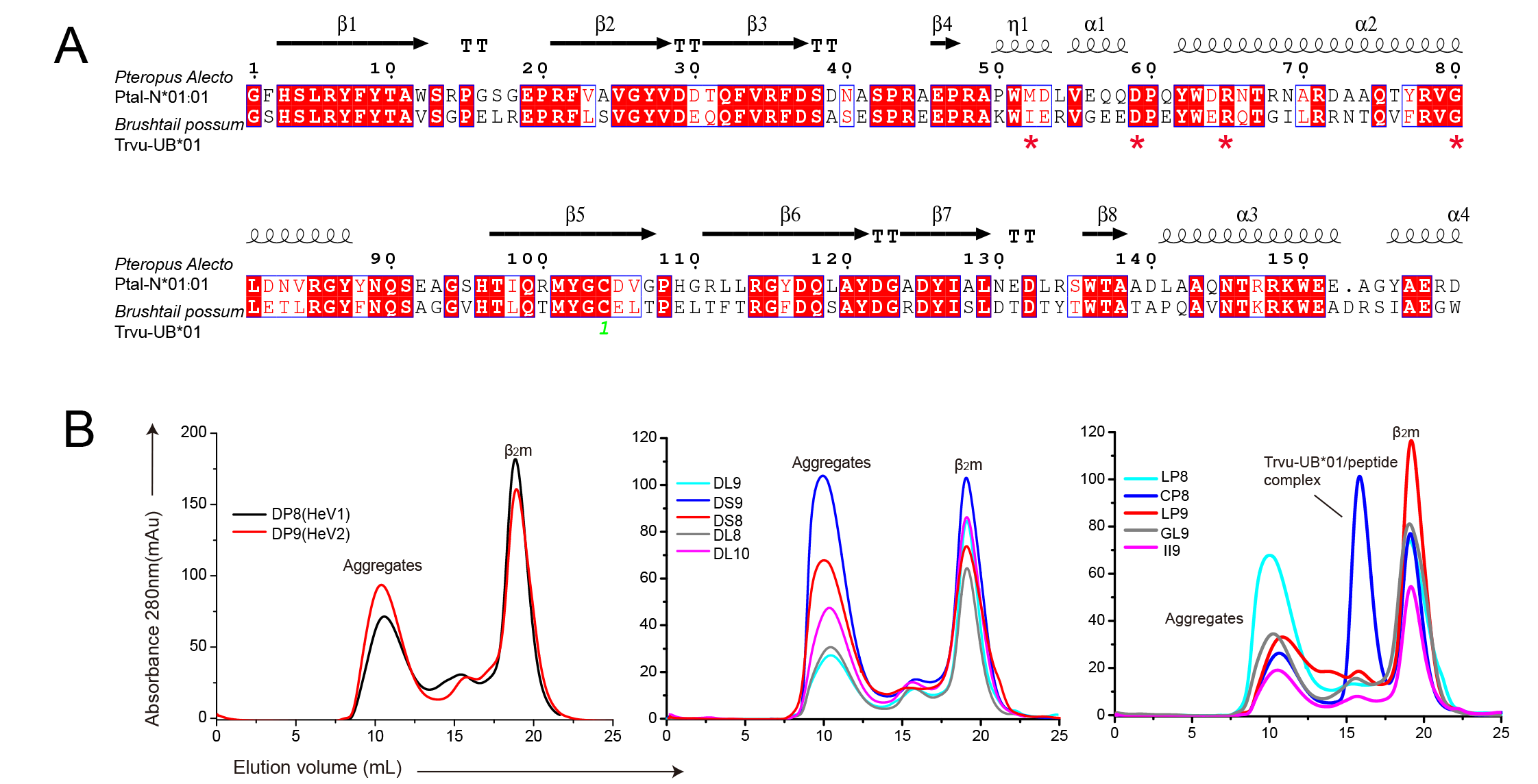

Supplement: S7 Fig — (A) Structure-based sequence alignment of Ptal-N*01:01 and Trvu-UB*01. (B-C) The peptide predictions refer to pocket features. The binding of peptides derived from possum nidovirus with Trvu-UB*01 were evaluated by co-refolding; the peptide sequence is listed. (TIF) [file pbio.3000436.s007.tif]
