# Supplementary material for: Peptide presentation by bat MHC class I provides new insight into the antiviral immunity of bats
Source: PLoS Biol. 2019 Sep 9;17(9):e3000436. doi: 10.1371/journal.pbio.3000436 (PMC6752855; doi:10.1371/journal.pbio.3000436)
Supplement: S1 Table — Raw data corresponding to Fig 1. (DOCX) [file pbio.3000436.s008.docx]

| Species | Version | Insert | 59AA | 65AA |
| --- | --- | --- | --- | --- |
| Ptal-N*0101 | AMD11115.1 | 3 | D | R |
| Pteropus_vampyrus | XP_011384364.1 | 3 | D | R |
|  | XP_023382220.1 | 3 | D | R |
|  | XP_011384363.1 | 3 | D | R |
|  | XP_023382219.1 | 3 | D | R |
|  | XP_011384364.1 | 3 | D | R |
|  | XM_011386062.2 | 3 | D | R |
|  | XM_011387637.1 | 3 | E | R |
| Rousettus_aegyptiacus | XM_016142773.1 | 3 | D | R |
|  | XM_016137085.1 | 3 | D | Q |
|  | XM_016135309.1 | 3 | D | Q |
|  | XM_016164885.1 | 3 | D | Q |
|  | XM_016137266.1 | 3 | D | Q |
|  | XM_016146443.1 | 3 | D | E |
|  | XP_015992346.1 | 3 | D | R |
|  | XP_015998259.1 | 3 | D | R |
|  | XP_015986808.1 | 3 | D | R |
| Eptesicus_fuscus | XP_008137465.1 | 0 | E | L |
|  | XP_008159910.1 | 5 | Y | E |
| Hipposideros_armiger | XP_019482266.1 | 5 | L | R |
|  | XP_019490480.1 | 5 | L | E |
|  | XP_019488329.1 | 5 | D | R |
|  | XP_019488328.1 | 5 | D | R |
|  | XP_019489700.1 | 0 | E | R |
|  | XP_019489562.1 | 5 | L | Q |
| Myotis_brandtii | XP_014406021.1 | 0 | E | Q |
|  | XP_005858976.1 | 5 | D | R |
|  | XP_014386456.1 | 5 | D | R |
|  | XP_014392898.1 | 5 | Y | E |
|  | EPQ18390.1 | 5 | R | E |
|  | XP_014392893.1 | 5 | Y | E |
| Myotis_lucifugus | XP_014306391.1 | 5 | Y | R |
|  | XP_006107251.2 | 5 | Y | R |
|  | XP_006107180.2 | 5 | D | R |
|  | XP_023604980.1 | 5 | Y | E |
|  | XP_023603595.1 | 0 | E | L |
|  | XP_006107250.1 | 5 | D | E |
|  | XP_014306737.1 | 5 | R | R |
|  | XP_023603922.1 | 5 | D | R |
|  | XP_023604095.1 | 0 | E | L |
|  | XP_006103427.2 | 5 | R | G |
|  | XP_006108085.1 | 5 | Y | R |
|  | XP_023603914.1 | 5 | D | E |
|  | XP_023603691.1 | 5 | D | R |
|  | XP_014304659.1 | 5 | D | R |
|  | XP_006107466.1 | 5 | Y | E |
|  | XP_023603913.1 | 5 | D | E |
|  | XP_014304881.1 | 5 | D | E |
|  | XP_014304470.1 | 5 | D | E |
|  | XP_023603911.1 | 5 | D | E |
|  | XP_023603897.1 | 5 | D | E |
|  | XP_014304469.1 | 5 | D | E |
|  | XP_014304594.1 | 0 | E | L |
|  | XP_014304476.1 | 5 | D | R |
| Rhinolophus_sinicus | XP_019575440.1 | 0 | G | A |
| Desmodus_rotundus | XP_024426421.1 | 5 | H | Q |
| Opossum | AGA94630.1 | 3 | D | Q |
|  | AGK44870.1 | 3 | D | Q |
|  | AGK44869.1 | 3 | D | R |
|  | AGK44868.1 | 3 | D | G |
|  | AGK44867.1 | 3 | D | Q |
|  | AGK44865.1 | 3 | D | Q |
|  | AGK44864.1 | 3 | D | Q |
|  | ABA54230.1 | 3 | - | R |
|  | ABA54240.1 | 3 | - | R |
|  | ABA54239.1 | 3 | - | R |
|  | ACK43880.1 | 3 | D | R |
|  | ACK43877.1 | 3 | D | R |
|  | ACK43878.1 | 3 | D | R |
|  | ACK43881.1 | 3 | D | R |
|  | XP_007483505.1 | 3 | D | R |
|  | XP_007483506.1 | 3 | D | R |
|  | XP_016284336.1 | 0 | M | L |
|  | XP_016284335.1 | 0 | M | L |
|  | NP_001165306.1 | 0 | M | L |
|  | XP_007483513.1 | 3 | - | G |
|  | XP_016284338.1 | 3 | D | K |
|  | NP_001233176.1 | 3 | D | K |
|  | XP_007483503.1 | 0 | T | R |
| Tammar wallaby | Maeu-1*01 | 3 | D | R |
|  | Maeu-1*02 | 3 | D | R |
|  | Maeu-1*03 | 3 | E | L |
|  | Maeu-I*07 | 3 | D | R |
|  | Maeu-I*08 | 3 | D | R |
|  | Maeu-I*09 | 3 | D | R |
|  | Maeu-I*10 | 3 | D | R |
|  | Maeu-I*11 | 3 | D | R |
|  | Maeu-I*12 | 3 | D | R |
|  | Maeu-I*13 | 3 | D | R |
|  | Maeu-I*14 | 3 | E | R |
|  | Maeu-I*15 | 3 | E | R |
|  | Maeu-I*16 | 3 | E | R |
|  | Maeu-I*17 | 3 | E | R |
| Koala | Phci-UA*01:01 | 3 | E | Q |
|  | Phci-UA*02:01 | 3 | E | Q |
|  | Phci-UA*03:01 | 3 | E | Q |
|  | Phci-UA*04:01 | 3 | E | R |
|  | Phci-UA*05:01 | 3 | E | Q |
|  | Phci-UA*06:01 | 3 | E | Q |
|  | Phci-UA*07:01 | 3 | E | Q |
|  | Phci-UB*01:01 | 3 | E | Q |
|  | Phci-UB*02:01 | 3 | E | Q |
|  | Phci-UB*01:02 | 3 | E | Q |
|  | Phci-UB*04:01 | 3 | E | R |
|  | Phci-UB*03:01 | 3 | E | R |
|  | Phci-UC*01:01 | -3 | - | R |
|  | Phci-UC*02:01 | -3 | - | R |
|  | Phci-UC*03:01 | -3 | - | R |
|  | Phci-UD*01:01 | 3 | D | R |
|  | Phci-UE*01:01 | -3 | - | R |
|  | Phci-UE*01:02 | -3 | - | R |
|  | Phci-UH*01:01 | 3 | E | R |
|  | Phci-UH*01:02 | 3 | E | R |
|  | Phci-UH*01:03 | 3 | E | R |
|  | Phci-UI*01:01 | -3 | E | R |
|  | Phci-UI*02:01 | -3 | E | R |
|  | Phci-UI*01:02 | -3 | E | R |
|  | Phci-UF*01:01 | -3 | I | R |
|  | Phci-UG*01:01 | 3 | D | R |
|  | Phci-UJ*01:01 | -3 | D | R |
|  | Phci-UK*01:01 | -3 | - | R |
| Tasmanian devil | Saha-I*01 | 3 | D | Q |
|  | Saha-I*12 | 3 | D | R |
|  | Saha-I*13 | 3 | D | R |
|  | Saha-UA | 3 | D | Q |
|  | Saha-UB | 3 | D | R |
|  | Saha-UC | 3 | D | R |
| Platypus | Oran-1-1 | 1 | V | D |
|  | Oran-1-2 | 1 | G | Q |
|  | Oran-2-1 | 1 | G | R |
