# Supplementary material for: Peptide presentation by bat MHC class I provides new insight into the antiviral immunity of bats
Source: PLoS Biol. 2019 Sep 9;17(9):e3000436. doi: 10.1371/journal.pbio.3000436 (PMC6752855; doi:10.1371/journal.pbio.3000436)
Supplement: S2 Table — Raw data corresponding to Fig 2. (DOCX) [file pbio.3000436.s009.docx]

| Name | Derived protein | Position | Sequence | Binding Score^a^ | Total Score^b^ | Refolding^c^ | |
| --- | --- | --- | --- | --- | --- | --- | --- |
| **HeV1**^d^ | Phosphoprotein^d^ | 481-488 | **DFANTFLP** | 0.001876 | -335.539 | | +++ |
| **HeV2** | fusion protein | 177–185 | **DYINTNVLP** | 0.013842 | -357.978 | | + |
| EBOV-GP1 | GP | 26-34 | LFQRTFSIP | 0.001081 | -317.356 | | - |
| EBOV-GP2 | GP | 136-146 | RYVHKVSGTGP | 0.000418 | -326.242 | | - |
| EBOV-GP3 | GP | 152-161 | AFHKEGAFFL | 0.051231 | -324.529 | | - |
| EBOV-GP4 | GP | 162-170 | LYDRLASTV | 0.075827 | -356.754 | | - |
| EBOV-GP5 | GP | 192-202 | DFFSSHPLREP | 0.000539 | -329.055 | | ++ |
| **EBOV-NP1**^e^ | NP | 65-74 | **DFQESADSFL** | 0.053761 | -331.738 | | +++ |
| **EBOV-NP2** | NP | 65-75 | **DFQESADSFLL** | 0.015147 | -341.414 | | +++ |
| EBOV-NP3 | NP | 65-76 | DFQESADSFLLM | /^f^ | -327.001 | | ++ |
| EBOV-NP4 | NP | 65-77 | DFQESADSFLLML | / | -322.562 | | ++ |
| **MERS-CoV-S1** | S | 384-393 | **DFSPLLSGTP** | 0.001362 | -347.757 | | ++ |
| MERS-CoV-S2 | S | 384-394 | DFSPLLSGTPP | / | -322.262 | | + |
| MERS-CoV-S3 | S | 422-430 | DFTCSQISP | 0.001778 | -319.248 | | +++ |
| MERS-CoV-S4 | S | 522-531 | QYSPCVSIVP | 0.001654 | -335.884 | | + |
| MERS-CoV-S5 | S | 539-547 | DYYRKQLSP | 0.008682 | -358.005 | | + |
| MERS-CoV-S6 | S | 539-548 | DYYRKQLSPL | 0.021122 | -350.359 | | + |
| MERS-CoV-S7 | S | 868-876 | DFNLTLLEP | 0.014050 | -352.338 | | +++ |
| **H17N10-NP** | NP | 289-297 | **DFEKEGYSL** | 0.166936 | -362.967 | | ++ |
| H17N10-PB1-1 | PB1 | 155-163 | YKLNESGRL | 0.035445 | -347.070 | | - |
| H17N10-PB1-2 | PB1 | 739-753 | DFSEITNTCKAIEAL | / | -311.350 | | - |
| H17N10-PB2-1 | PB2 | 114-122 | VYKMYFDRL | 0.040486 | -349.355 | | - |
| H17N10-PB2-2 | PB2 | 117-125 | MYFDRLERL | 0.256357 | -349.002 | | - |
| H18N11-PB1-1 | PB1 | 155-163 | YTANESGRL | 0.218380 | -344.038 | | - |
| H18N11-PB1-2 | PB1 | 619-627 | EYRGRLCNP | 0.001089 | -346.518 | | - |
| H18N11-PB1-3 | PB1 | 739-753 | DFSEIMNICKAIENL | / | -315.694 | | - |

^a^ Calculated by using <http://www.cbs.dtu.dk/services/NetMHCpan/>.

^b^ Calculated by using structure-based prediction program <http://flexpepdock.furmanlab.cs.huji.ac.il/>.
^c^ Peptides that can help the Ptal-N*01:01 H chain renature with bat β_2_m are marked as +, otherwise -.The number of “+” represents the degree of renaturation( ≥100mAu “++”, ≥200mAu “+++” ).
^d^ Protein resource: HeV/Australia/Horse/1994/Hendra strain; Ebola virus nucleocapsid protein (NP) (GenBank: AF054908.1); Zaire ebolavirus (GP) (GenBank: AKG65250.1); Middle East respiratory syndrome-related coronavirus S protein (GenBank: AXN92228.1); H17N10 influenza-like virus (A/little yellow-shouldered bat/Guatemala/060/2010(H17N10)); H18N11 influenza-like virus (A/flat-faced bat/Peru/033/2010(H18N11)).

^e^ The peptides that had been analyzed in this article are denoted with bold letters.

^f^ Scoring for peptides with residues 10 or more is not available.
