# Supplementary material for: Peptide presentation by bat MHC class I provides new insight into the antiviral immunity of bats
Source: PLoS Biol. 2019 Sep 9;17(9):e3000436. doi: 10.1371/journal.pbio.3000436 (PMC6752855; doi:10.1371/journal.pbio.3000436)
Supplement: S3 Table — Peptides’ information corresponding to Fig 4. HeV, Hendra virus. (DOCX) [file pbio.3000436.s010.docx]

| Name | Derived protein | Position | Sequence | Refolding^a^ | |  |
| --- | --- | --- | --- | --- | --- | --- |
| HeV1  HeV1-D1A  HeV1-F2A  HeV1-T5A  HeV1-P8A | Phosphoprotein^b^ | 481-488  -  -  -  - | DFANTFLP  **A**^d^FANTFLP  D**A**ANTFLP  DFAN**A**FLP  DFANTFL**A** | **+++**  **++**  **+**  **+++**  **++** |  |  |
| HeV2  HeV2-D1A  HeV2-Y2A  HeV2-N6A  HeV2-P9A | fusion protein^c^ | 177–185  -  -  -  - | DYINTNVLP  **A**YINTNVLP  D**A**INTNVLP  DYINT**A**VLP  DYINTNVL**A** | **+++**  **-**  **+**  **+++**  **-** | | |

^a^Peptides that can help the Ptal-N*01:01 H chain renature with bat β_2_m are marked as +, otherwise -. The number of “+” represents the degree of renaturation( ≥100mAu“++”, ≥200mAu“+++” ).
^b^The virus stain: HeV/Australia/Horse/1994/Hendra strain.
^c^The mutant residues positions of peptides are denoted with underlined bold letters.
