# Supplementary material for: Peptide presentation by bat MHC class I provides new insight into the antiviral immunity of bats
Source: PLoS Biol. 2019 Sep 9;17(9):e3000436. doi: 10.1371/journal.pbio.3000436 (PMC6752855; doi:10.1371/journal.pbio.3000436)
Supplement: S4 Table — (DOCX) [file pbio.3000436.s011.docx]

| Name | Peptide resource | Sequence | Refolding^a^ |
| --- | --- | --- | --- |
| Bat1 | Translation initiation factor eIF-2B subunit alpha | GVLTPSAVSDEL | - |
| Bat2 | DNA replication licensing factor MCM6 | PYLVVNPNYLLED | - |
| Bat3 | Trifunctional enzyme subunit alpha, mitochondrial | LIDHANSPTKKFY | - |
| Bat4 | UbiA prenyltransferase domain-containing protein 1 | GIFLAPAGSLPKL | - |
| Bat5 | Small nuclear ribonucleoprotein F | YIDGALSGHLGEVL | - |
| Bat6 | Trafficking protein particle complex subunit 10 | AVGVFASGSPLSIL | - |
| Bat7 | Ptal-N*01:01 | GFHSLRYFYTAWSRP | - |
| Bat8 | RNA-binding protein with multiple splicing | VTYQPSADQQRELPG | - |
| Bat9 | Vacuolar-sorting protein SNF8 | YSQEITAEEAREALP | - |
| Bat10 | Vitamin K epoxide reductase complex subunit 1 | NVRDVQGPQDKVKGH | - |
| Bat11 | Heat shock protein HSP 90-beta | RELISNASDALDKIR | - |
| Bat12 | Prenylcysteine oxidase | RVNYGQSTNINGFVG | - |
| Bat13 | Proteasome activator complex subunit 2 | EKIVNPKGEEKPSMY | - |
| Bat14 | 60 kDa heat shock protein, mitochondrial | KIMQSSSEVGYDAM | - |
| Bat15 | Ptal-N*01:01 | DLEQKLISEEDLI | - |
| Bat16 | Ptal-N*01:01 | EEDLEQKLISEED | - |
| Bat17 | Ptal-N*01:01 | EDLEQKLISEEDLI | - |
| Bat18 | Ptal-N*01:01 | EEDLEQKLISEEDL | - |
| Bat19 | Ptal-N*01:01 | EEDLEQKLISEEDLI | - |
| Bat20 | Ptal-N*01:01 | VETRPDGNGAFQKW | - |

^a^peptides that can help the Ptal-N*01:01 H chain renature with bat β_2_m are marked as +, otherwise -.
