# Supplementary material for: Peptide presentation by bat MHC class I provides new insight into the antiviral immunity of bats
Source: PLoS Biol. 2019 Sep 9;17(9):e3000436. doi: 10.1371/journal.pbio.3000436 (PMC6752855; doi:10.1371/journal.pbio.3000436)
Supplement: S5 Table — (DOCX) [file pbio.3000436.s012.docx]

| Name | Derived protein | Position | Sequence | Refolding^a^ |  |
| --- | --- | --- | --- | --- | --- |
| HeV1 | phosphoprotein | 481-488 | DFANTFLP | + | |
| HeV1-N2 |  |  | SDDFANTFLP | - |  |
| MERS-CoV-S7 | S | 422-430 | DFNLTLLEP | + |  |
| MERS-CoV-S7-N2 |  |  | GGDFNLTLLEP | - |  |
| MERS-CoV-S7-N4 |  |  | GFGGDFNLTLLEP | - |  |
| MERS-CoV-S7-N6 |  |  | IPGFGGDFNLTLLEP | - |  |

| EBOV-NP1 | NP | 65-74 | DFQESADSFL | + |
| --- | --- | --- | --- | --- |
| EBOV-NP1-N2 |  |  | GVDFQESADSFL | - |

^a^Peptides that can help the Ptal-N*01:01 H chain renature with bat β_2_m are marked as +, otherwise -.
