# Supplementary material for: Peptide presentation by bat MHC class I provides new insight into the antiviral immunity of bats
Source: PLoS Biol. 2019 Sep 9;17(9):e3000436. doi: 10.1371/journal.pbio.3000436 (PMC6752855; doi:10.1371/journal.pbio.3000436)
Supplement: S2 Data — (ZIP) [file pbio.3000436.s014.zip › SARS-MERS CODE/Software and code.docx]

Software and code

Data collection:

The proteomes of MERS-CoV genomes and SARS-CoV genomes were retrieved from GenBank, respectively.

Data analysis:

The sequences of MERS-CoV and 1000 SARS-CoV alignment with MAFFT, the dominant amino acid for each site was elected as a reference sequence , in order to find the number of overall mutations for each amino acid, the computer code is:

import java.io.*;

import java.util.*;

public class AminoAcidMutation{

public AminoAcidMutation(){}

//return the list of file name in the folder

public File[] ReadFolder(String folderpath){

File[] filelist = new File[0];

try{

File folderToRead = new File(folderpath);

if(folderToRead.isDirectory()){

filelist = folderToRead.listFiles();

}

}catch(NullPointerException e){}

return filelist;

}

public Vector getNameAndSeq(File filename){

Vector all = new Vector(1);

Vector name = new Vector(1);

Vector seq = new Vector(1);

try{

BufferedReader in = new BufferedReader(new FileReader(filename));

String str;

StringBuffer sb_name = new StringBuffer();

StringBuffer sb_seq = new StringBuffer();

while((str=in.readLine())!=null){

if(str.startsWith(">")){

if(sb_name.length()>0&&sb_seq.length()>0){

if(!name.contains(sb_name.toString())){

name.add(sb_name.toString());

seq.add(sb_seq.toString());

}

sb_name.delete(0,sb_name.length());

sb_seq.delete(0,sb_seq.length());

}else{

if(sb_name.length()>0){

System.out.println(sb_name.toString());

sb_name.delete(0,sb_name.length());

}

if(sb_seq.length()>0){

sb_seq.delete(0,sb_seq.length());

}

}

sb_name.append(str.substring(1));

}else{

sb_seq.append(str);

}

}

if(sb_name.length()>0&&sb_seq.length()>0){

name.add(sb_name.toString());

seq.add(sb_seq.toString());

}

in.close();

}catch(IOException e){}

all.add(name);

all.add(seq);

return all;

}

public static void main(String [] args){

AminoAcidMutation aam = new AminoAcidMutation();

File[] fileList = aam.ReadFolder(args[0]);

Vector aa = new Vector(1);

aa.add("A");aa.add("R");aa.add("N");aa.add("D");aa.add("C");aa.add("Q");aa.add("E");

aa.add("G");aa.add("H");aa.add("I");aa.add("L");aa.add("K");aa.add("M");aa.add("F");

aa.add("P");aa.add("S");aa.add("T");aa.add("W");aa.add("Y");aa.add("V");

int[] mutationNum = new int[aa.size()];

int[] totalNum = new int[aa.size()];

for(int i=0;i<fileList.length;i++){

Vector nameAndSeq = aam.getNameAndSeq(fileList[i]);

Vector seq = (Vector)nameAndSeq.get(1);

for(int j=0;j<seq.size()-1;j++){

for(int k=j+1;k<seq.size();k++){

String seq_1 = (String)seq.get(j);

String seq_2 = (String)seq.get(k);

for(int m=0;m<seq_1.length();m++){

String aa_1 = seq_1.substring(m,m+1);

String aa_2 = seq_2.substring(m,m+1);

int index_1 = aa.indexOf(aa_1);

int index_2 = aa.indexOf(aa_2);

if(index_1>-1){

totalNum[index_1]++;

}

if(index_2>-1){

totalNum[index_2]++;

}

if(!aa_1.equalsIgnoreCase(aa_2)){

if(index_1>-1){

mutationNum[index_1]++;

}

if(index_2>-1){

mutationNum[index_2]++;

}

}

}

}

}

}

try{

BufferedWriter out = new BufferedWriter(new FileWriter(args[1]));

for(int i=0;i<aa.size();i++){

out.write((String)aa.get(i)+" "+String.valueOf(mutationNum[i])+" "+String.valueOf(totalNum[i]));

out.newLine();

}

out.close();

}catch(IOException e){}

}

}
